# Two different sulfotransferases modify sugars of the N-linked tetrasaccharide decorating *Halobacterium salinarum* glycoproteins

**DOI:** 10.1128/mbio.03534-24

**Published:** 2025-02-25

**Authors:** Marianna Zaretsky, Zlata Vershinin, Lihi Erez, Iris Grossman-Haham, Jerry Eichler

**Affiliations:** 1 Department of Life Sciences, Ben-Gurion University of the Negev, Beersheva, Israel; 2 The Ilse Katz Institute for Nanoscale Science and Technology, Ben-Gurion University of the Negev, Beersheva, Israel; Albert-Ludwigs-Universitat Freiburg, Freiburg, Germany

**Keywords:** archaea, *Halobacterium salinarum*, glycoprotein, N-glycosylation, sulfotransferase

## Abstract

Despite providing the first example of archaeal N-glycosylation almost 50 years ago, detailed insight into the pathway used by *Halobacterium salinarum* to assemble and attach an N-linked tetrasaccharide decorating glycoproteins in this haloarchaea has only recently appeared. Still, numerous components of this pathway remain to be identified, including sulfotransferase(s), which modify the third and fourth tetrasaccharide sugars. In the present report, a series of bioinformatics, genetic, biochemical, and structural approaches served to reveal how membrane-associated VNG1056C and soluble VNG1057C respectively sulfate the iduronic acid at tetrasaccharide position three and the terminal glucuronic acid, seemingly independent of each other. Deletion of *VNG1056C* but not of *VNG1057C* reduced cell motility to a minor degree and did not cause archaellum filament bundling. Finally, transcription of *VNG1056C* or *VNG1057C* was augmented upon deletion of the other when cells were grown in low but not high salinity conditions possibly in an attempt to compensate for the loss of sugar sulfation resulting from the deletion. This augmented transcription, however, had no effect on the extent of tetrasaccharide sulfation. With demonstrated roles in *Hbt. salinarum* N-glycosylation, VNG1056C and VNG1057C were respectively re-annotated as Agl30 and Agl31, employing the nomenclature used to define archaeal N-glycosylation pathway components.

**IMPORTANCE:**

Like essentially all Archaea, the halophile *Halobacterium salinarum* performs N-glycosylation, namely, the covalent attachment of a glycan moiety to select asparagine residues in a target protein. Moreover, *Hbt. salinarum* represents one of the few current archaeal examples in which the pathway of N-glycosylation has been largely defined. Still, several components of this pathway remain to be defined, including the sulfotransferase(s) responsible for modifying the iduronic acid and glucuronic acid corresponding to the third and final sugars of the N-linked tetrasaccharide that decorates glycoproteins in this haloarchaeon. In the present report, a series of bioinformatics, genetic, biochemical, and structural approaches served to reveal how membrane-associated VNG1056C and soluble VNG1057C respectively sulfate the iduronic acid at tetrasaccharide position three and the terminal glucuronic acid, seemingly independent of each other. The need for two different enzymes reflects the sulfation of these sugars at distinct positions.

## INTRODUCTION

*Halobacterium salinarum* are halophilic archaea that grow in NaCl concentrations near or at saturation (1). In addition to their ability to survive in such hyper-saline conditions, *Hbt. salinarum* have also provided numerous examples of biological novelty. For instance, *Hbt. salinarum* offered the first example of non-eukaryal protein glycosylation, with the surface (S)-layer glycoprotein that comprises the protein shell surrounding the cell being both N- and O-glycosylated (2). Soon after, archaellins, the building blocks of the archaeal swimming device, the archaellum (3), were also shown to be N-glycosylated (4). Biochemical studies conducted at the time provided an initial description of N-linked glycans decorating *Hbt. salinarum* glycoproteins and defined selected steps in the N-glycosylation process (for review, see [5]). More recently, *Hbt. salinarum* N-glycosylation has been re-visited using a variety of bioinformatics, genetic, biochemical, and structural tools and approaches (6–10). Nuclear magnetic resonance (NMR) spectroscopy was used to define the composition of the N-linked tetrasaccharide decorating *Hbt. salinarum* glycoproteins as β-D-GlcA(2S)-(1→4)-α-L-IdoA(3S)-(1→4)-β-D-GlcA-(1→4)-β-D-Glc-Asn, where Glc is glucose; IdoA is iduronic acid; GlcA is glucuronic acid; and S refers to sulfation (9). Moreover, much of the pathway used to assemble and attach this glycan was delineated by a strategy combining gene deletions and mass spectrometry. In this manner, Agl28, Agl25, Agl26, and Agl27 were identified as the glycosyltransferases responsible for the sequential addition of the four N-linked tetrasaccharide sugars to a common dolichol phosphate (DolP) carrier. Agl29 was identified as the flippase that translocates the DolP-bound tetrasaccharide across the membrane (or contributes to such activity), and AglB was shown to be the oligosaccharyltransferase that transfers this glycan from its lipid carrier to target proteins (7, 8, 10). Still, several components of the *Hbt. salinarum* pathway used to generate the N-linked glycan remain to be defined, including the sulfotransferase(s) responsible for the modification of the IdoA and GlcA that respectively correspond to tetrasaccharide sugars three and four.

Despite not knowing which enzyme(s) sulfate the N-linked tetrasaccharide in *Hbt. salinarum*, earlier efforts, nonetheless, provided some insight into the process involved. It was revealed that tetrasaccharide sulfation occurs at the DolP-bound stage, rather than after the glycan has been transferred to the target protein (11, 12). Moreover, it seems that sulfation only occurs once the complete DolP-bound tetrasaccharide has been assembled since no such modification was detected in mutant strains only able to assemble tetrasaccharide precursors (8, 10). The most striking finding of previous studies of tetrasaccharide sulfation was that sugar three, namely, IdoA, was determined to be sulfated at the O-3 position (9). IdoA, an epimer of GlcA, is best known as a component of the eukaryal glycosaminoglycans (GAGs) heparin and heparin sulfate (13–15). In these structures, IdoA is sulfated at the O-2 position. While the rare O-3 sulfation of heparan sulfate glucosamines (16) and chondroitin sulfate GlcA has been reported (17), O-3 sulfation of IdoA, and, indeed, the inclusion of IdoA in an N-linked glycan is, to our knowledge, restricted to *Hbt. salinarum* (9, 18). Such sulfation may thus represent an aspect of protein glycosylation unique to Archaea. In contrast, the GlcA at the non-reducing end of the N-linked tetrasaccharide is sulfated at the O-2 position (9). Like the sulfotransferase(s) responsible, the order of IdoA and GlcA sulfation, as well as the physiological significance of these sulfation events, are not known.

In the present study, a series of bioinformatics, genetic, biochemical, structural, and cell biology approaches was used to answer these and other outstanding questions concerning the sulfation of sugars comprising the N-linked tetrasaccharide decorating *Hbt. salinarum* glycoproteins, including defining the two sulfotransferases responsible for this sugar modification.

## MATERIALS AND METHODS

### Cell growth

*Hbt. salinarum* NRC-1 parent and mutant strain cells were grown in a medium containing 250 g NaCl, 20 g MgSO_4_⋅7H_2_O, 3 g sodium citrate, 2 g KCl, and 10 g peptone per liter supplemented with 50 µg/mL uracil at 42°C (7, 19). *Haloferax volcanii* WR536 (H53) strain cells were grown in complete medium containing 206g NaCl, 39g MgSO_4_·7 H_2_O, 3 g yeast extract, 5 g tryptone, 1 mM MnCl_2_, 4 mM KCl, 3 mM CaCl_2_, and 50 mM Tris–HCl, pH 7.2, at 42°C (20). Novobiocin (1 µg/mL) was added to the growth medium of plasmid-transformed cells.

### Bioinformatics

BLAST searches using VNG1056C (UniProt entry Q9HQQ3), VNG1057C (UniProt entry Q9HQQ2), and VNG1063H (UniProt entry Q9HQP7) protein sequences as bait were performed at the UniProt site (https://www.uniprot.org/blast/) in October 2024 using the default UniProtKB reference proteomes + Swiss Prot as the target database, as well as other default settings. InterPro (https://www.ebi.ac.uk/interpro/) was consulted in October 2024 using default settings to determine whether VNG1056C, VNG1057C, and VNG1063H could be assigned to known protein families or detect the presence of domains or other important sites that could provide insight in protein function. The determination of the best structural homologues of VNG1056C, VNG1057C, and VNG1063H was performed by comparing the AlphaFold computational modeling tool-generated predicted structures (21) of each protein at the UniProt site (https://www.uniprot.org) against entries in the Protein Data Bank using the DALI protein structure comparison server (http://ekhidna2.biocenter.helsinki.fi/dali/ [22]; performed June 2024).

### Reverse transcriptase PCR and quantitative (q)PCR

Reverse transcriptase PCR was performed as described previously (7) using primers listed in Table 1. To quantify mRNA levels, qRT-PCR was performed. RNA was isolated from culture aliquots (1 mL) using an RNeasy Mini Kit (Qiagen) according to the manufacturer’s instructions. Contaminating DNA in the RNA samples was eliminated with RNase-Free DNase Set (Qiagen) during RNA extraction. RNA concentration was determined spectrophotometrically using a Nanodrop. Single-stranded cDNA was prepared from the extracted RNA using random hexameric primers in a Superscript IV 1st Strand System (Invitrogen). Relative transcript levels were then determined by qPCR analysis using a CFX384TM Real-time System (Bio Rad). The reaction mix contained 5 µL of SYBR green mix (Applied Biosystem), 0.3 µM of primers (listed in Table 1), 5 ng cDNA, and distilled deionized water in a total reaction volume of 10 µL. The following parameters were used: 95°C for 3 min, 40 cycles of 15 s at 95°C, and 1 min at 60°C for annealing, extension, and read fluorescence, respectively. Melting curve analysis was performed after each run to ensure the specificity of the products. The efficiency of each primer set was calculated using five-to-six serial dilutions of the parent sample. Using this efficiency value for each primer set, relative expression was calculated using the standard 2^−∆∆ct^ formula, with the *VNG0657G* housekeeping gene as a reference (23).

**TABLE 1:** Primers used in this study

Primer	Sequence
Deletion primers	
*VNG1056* deletion	
1056-up-Hind-F new	CGAGCAGACGCATCTGGATCCACGAAGCTTAGGACTTCATGCTGATGCTCGGG
rev-1056 up-new	CCGCGTTCGGTGGCAGTGACTTAGCATCGCTCGTTGTTGATCACTCA
Fr 1056 down-new	TGAGTGATCAACAACGAGCGATGCTAAGTCACTGCCACCGAACGCGG
1056-down-R-Nco-new	AGGTATCTAGAACCGGTGACGTCACCATGGAGTCCACAGGTAACGAACG
*VNG1057* deletion	
VNG1057-up-Hind-F	CGAGCAGACGCATCTGGATCCACGAAGCTTCTGCAATGATATGTCGCGCCG
VNG1057-down-F	GTTGGTAGGGCCCGCTATGCTGATGGTCCGCGTTCG
VNG1057-up-R	CGAACGCGGACCATCAGCATAGCGGGCCCTACCAAC
VNG1057-down-Nco-R	AGGTATCTAGAACCGGTGACGTCACCATGGCGGGCAATCACCGACAGAACG
*VNG1056,1057* deletion	
1056-up-Hind-F new	CGAGCAGACGCATCTGGATCCACGAAGCTTAGGACTTCATGCTGATGCTCGGG
Fr 1057 down new	GTGATCAACAACGAGCGATGCCCTACCAACTTCTCTGACTTAG
Rev 1056 up new	CTAAGTCAGAGAAGTTGGTAGGGCATCGCTCGTTGTTGATCAC
Rev 1057 down new Nco	AGGTATCTAGAACCGGTGACGTCACCATGGGACTGGATTTTTCTGCAATGATATG
*VNG1063* deletion	
Fr 1063 up Hind new	CGAGCAGACGCATCTGGATCCACGAAGCTTTACGCCGACCCAGAGTACTAC
Rev 1063 up new	GATGTCAGAGGTTGGGTTACTCGCATAGTGTATCGACCCAATTAG
Fr 1063 down new	CTAATTGGGTCGATACACTATGCGAGTAACCCAACCTCTGACATC
Rev 1063 down Nco new	AGGTATCTAGAACCGGTGACGTCACCATGGATGGGGCTACCGGTAGAAC
Validation primers	
fr-pNKB07-seq	TGTCACAGACGACGCTCCCGCA
rev-pNBK07-seq	GTTGGGTAACGCCAGGGTTTTC
fw-1055-FL	ATGCAAGCAGTCGTGCTCGCGG
rev-1055-FL	TCACTCGTTGCTCACTGCGTCG
fr-pRV-1058-Nde-Gibson	CACATTCGCGGACCTATTGCGCATATGCCGTCTAGTTCGAACCGCCATC
rev-pRV-1058-EcoRI-Gibson	ACCGTCTCGTGACAGCCGAATTCTCAGTGTAGGTCTACGTTCGGAATGTC
fw-1062-FL	ATGCCTGATTCCCCGTTCGTATC
rev-1062-FL	TCAGGTCGTGTCTCCGGATAC
fw-1064-FL	ATGCACGCCACAATCGAC
rev-1064-FL	CTAGAGTTTGCCTCTGGTGCTC
VNG1063H FW1 VNG1063H REV1	GGAGCGGGATACGGAAC ACTCTATGTAGCCGAGTTCTTTG
qPCR primers	
VNG0657G FW	CGGATTCGGTCGAGTTTCAT
VNG0657G Rev	CACATCGTGGTGATCCAGTT
VNG1056C FW	GGACTTTGGACACCTGGATATG
VNG1056C Rev	AACCGTTCTGTCGGTGATTG
VNG1057C FW	AAACGGTGCGGAGATCAAG
VNG1057C Rev	CTCGCAGCTTGGATGTAGTT
VNG1063H FW	CGTTCATTATTTCCAGCCACAC
VNG1063H Rev	CACACGTCTTCGTCGATCTT
Cloning primers	
fr-pRV-NdeI-1056	CACATTCGCGGACCTATTGCGCATATGGCGGGAAATCTCCGTGGC
rev-pRV-1056-EcoRI	ACCGTCTCGTGACAGCCGAATTCTTAATGATGATGATGATGATGCTTTTCG AATTGCGGGTGGCTCCACAAGTATCCGAGATCCTCGAGATG
fr-pRV-NdeI-1057	CACATTCGCGGACCTATTGCGCATATGGCTGAATCGGGTAGCGAC
rev-pRV-1057-EcoRI	ACCGTCTCGTGACAGCCGAATTCTTAATGATGATGATGATGATGCTTTTCG AATTGCGGGTGGCTCCACGTGTACCCCAACTCCTCCAG

### Gene deletions

*Hbt. salinarum* Δ*ura3* cells deleted of *VNG1056C*, *VNG1057C, VNG1063H*, or *VNG1056C*/*VNG1057C* were generated using a previously described double-crossover counter-selection method (7, 8, 24). Briefly, approximately 500 bp-long flanking regions upstream and downstream of the target gene were PCR amplified and inserted into the *HindIII* and *NcoI* restriction site of plasmid pNBK07 (25) by isothermal assembly (26) to create plasmids pNBK07ko1056, pNBK07ko1057, pNBK07ko1063, and pNBK07ko1056/57. Following Sanger sequencing, these plasmids were individually introduced into the Δ*ura3* strain and selected on solid medium (20 g/L agar plates) containing mevinolin (10 µg/mL). The resulting strains were then counter-selected on plates containing 5-fluoroorotic acid (300 µg/mL) and uracil to remove the integrated plasmid, yielding the deletion strains. All incubation steps during transformation and counter-selection were conducted at 42°C. Deletions were confirmed by PCR and qRT-PCR. Primers used are listed in Table 1.

### Liquid chromatography–electrospray ionization mass spectrometry (LC–ESI MS)

LC–ESI MS analysis of the *Hbt. salinarum* and *Hfx. volcanii* S-layer glycoproteins was performed essentially as previously described (8). Here, however, the peptides were analyzed using a Q Exactive HF mass spectrometer (Thermo) fitted with a capillary HPLC system (easy nLC 1200, Thermo Fisher, HF). The peptides were loaded in solvent A (0.1% formic acid in water) on a homemade capillary column (30 cm, 75 μm ID) packed with Reprosil C18-Aqua (Dr. Maisch HPLC, Ammerbuch, Germany). The peptide mixture was resolved with a 6 to 34% linear gradient of solvent B (80% acetonitrile with 0.1% formic acid) for 60 min, followed by a gradient of 15 min of 34 to 95% and 15 min at 95% solvent B at flow rates of 0.15 µL/min. Mass spectrometry was performed in a positive mode (m/z 300–1,500, resolution 60,000 for MS1 and 15,000 for MS2) using repetitive full MS scan, followed by high-collision dissociation (at 27 normalized collision energy) of the 18 most dominant ions (>1 charges) selected from the first MS scan. The AGC settings were 3 × 10^6^ for the full MS and 1 × 10^5^ for the MS/MS scans. The intensity threshold for triggering MS/MS analysis was 1.3 × 10^5^. A dynamic exclusion list was enabled with an exclusion duration of 20 s.

### Plate motility assay

Cell motility was assayed as previously described (8). Briefly, liquid cultures of parent and deletion stain cells were grown to a logarithmic phase (OD_600_, ~0.8), and aliquots (10 µL) were applied to the center of the plates containing a semi-solid medium containing 0.3% (wt/vol) agar. After 4 days at 42°C, the diameters of the motility halos on four or five plates per strain were measured.

### VNG1056C and VNG1057C expression in *Hfx. volcanii*

For the expressions of VNG1056C and VNG1057C in *Hfx. volcanii*, the pRV1-ptna (Hisx6, StrepII) plasmid (27) was used. Genes encoding VNG1056C and VNG1057C were amplified by PCR from *Hbt. salinarum* genomic DNA using primers containing *NdeI* and *EcoRI* restriction sites (listed in Table 1). The PCR products were ligated into plasmid pRV1-ptna cleaved with *NdeI* and *EcoRI* using a Gibson Assembly Kit (New England BioLabs) according to the manufacturer’s instructions. The resulting plasmids were introduced into *Hfx. volcanii* cells as previously described (28).

### Sub-cellular fractionation

Cytosolic and membrane protein fractionation was performed as previously described (29), with a few modifications. Briefly, 5 mL cultures of *Hfx. volcanii* transformed with plasmids pRV1-ptna-1056 or pRV1-ptna-1057 were grown to a logarithmic phase. The cultures were centrifuged, and the pellets were washed with 3.5 M basal salt solution (204.5 g NaCl, 20 g MgSO_4_·7H_2_O, 3 g sodium citrate, 2 g KCl per liter). The cells were resuspended with 1 mL 3.5 M basal salt solution containing 1 mM phenylmethylsulfonyl fluoride (Sigma-Aldrich) and broken by sonication (2 s on and 1 s off for 90 s, 25% output; Sonics VCX750). Remaining intact cells were cleared by centrifugation (9,000 × *g*, 10 min, 4°C), and the supernatant was centrifuged in an ultracentrifuge (240,000 × *g*, 12 min, 4°C; Sorvall M120). The resulting supernatant (cytosolic fraction) was precipitated with trichloroacetic acid (TCA), whereas the pelleted membrane fraction was first resuspended in 200 µL of distilled water, and then precipitated with TCA. The extracted proteins were resolved on SDS-PAGE gels, electrotransferred to a polyvinylidene fluoride membrane (BioRad), and probed with HRP-conjugated anti-StrepII (GenScript) or anti-SRP54 (30) antibodies. HRP-conjugated goat anti-rabbit antibodies (BioRad) served as secondary antibodies. The S-layer glycoprotein was identified by Coomassie staining (Expedeon, InstantBlue).

### Archaellum filament enrichment

*Hbt. salinarum* archaellum filaments were enriched from the spent growth medium of parent, Δ*VNG1056C*, Δ*VNG1057C*, and Δ*VNG1056C/*Δ*VNG1057C* strain cells as previously described (7).

### Transmission electron microscopy (TEM)

Enriched archaellum filaments were derived from 100 mL of culture (31). Pellets were resuspended in 3 mL of basal salt solution (250 g NaCl, 20 g MgSO_4_⋅7H_2_O, 3 g sodium citrate, 2 g KCl per liter) diluted four-fold in water. Three-microliter aliquots of each sample were applied to glow-discharged Quantifoil R 1.2/1.3 holey carbon grids (Quantifoil, Großlöbichau, Germany), which were then manually blotted for 4 s at room temperature and vitrified by rapid plunging into liquid ethane using a home-built plunging apparatus. The frozen samples were stored in liquid nitrogen until imaging. Imaging was performed using a Glacios microscope operated at 200 kV and equipped with a Falcon 4i direct electron detector coupled to a Selectris X energy filter (Thermo Fisher Scientific), as described (32). Negative-stain TEM imaging of cell-attached archaellum filaments was performed as described (32). Cells were imaged from two cultures grown independently.

### Electrostatic potential depiction

Electrostatic potential surface was depicted by mapping atomic Gasteiger charges onto the molecular van der Waals surface using Avogadro version 2.0 (33).

## RESULTS

### Bioinformatics-based approaches predict VNG1056C, VNG1057C, and VNG1063H to be sulfate-processing proteins

As a first step in identifying the sulfotransferase(s) responsible for the sulfation of the third and fourth sugars of the N-linked tetrasaccharide decorating *Hbt. salinarum* glycoproteins, genes within the *VNG1068G* (*aglB*)-anchored N-glycosylation gene cluster were considered, specifically, *VNG1056C, VNG1057C*, and *VNG1063H*, which are currently annotated as encoding alkaline phosphate domain-containing proteins (6). A BLAST search using VNG1056C as bait listed predicted haloarchaeal sulfatases or sulfatase domain-containing proteins within the top 10 hits (47–75% identity; scores of 749 to 1,365; E-values of 3.8e−95 to 0), while a similar search using VNG1057C as bait recognized predicted haloarchaeal sulfatases or arylsulfatases (39–43% identity; scores of 780 to 822; E-values of 3.8e−96 to 2e−102). At the same time, a BLAST search using VNG1063H as bait listed the predicted lipoteichoic acid synthase family protein from a haloarchaeon as the top hit (40% identity; score of 585; E-value of 8.2e−70), followed by haloarchaeal sulfatase-like hydrolases/transferases as the next top 10 hits with predicted functions (30–38% identity; scores of 291 to 335; E-values of 1.3e−26 to 8.1e−33). InterPro-based searches failed to assign either VNG1056C or VNG1063H to a protein family, while VNG1057C was recognized as an arylsulfatase family member (IRP050738). All three proteins were, however, assigned to the alkaline phosphatase-like core domain superfamily (IPR017850) that also includes sulfatases and N-sulfoglucosamine sulphohydrolases.

Structural homology analysis of the products of *VNG1056C*, *VNG1057C*, and *VNG1063H* was next performed to address whether any of these proteins correspond to sulfotransferases or related enzymes. To identify the closest structural homologue of the AlphaFold-generated model for each of the three *Hbt. salinarum* proteins, each predicted structure was compared with the experimental protein structures in the Protein Data Bank using the DALI server. This strategy deemed the *Bacteroides thetaiotaomicron* VPI-5482 2-O glycosaminoglycan sulfatase BT1596 (PDB 5G2U) as best resembling VNG1056C and VNG1063H (RMSD values of 3.0 and 3.1 Å, respectively) and the *Formosa agariphila* family S1_7 ulvan-specific sulfatase FA22070 (PDB 6HHM) as best resembling VNG1057C (RMSD value of 3.7 Å) at the structural level. The former acts as a uronic acid 2-O sulfatase (34), while the latter is a xylose 2-O sulfatase (35).

Together, these bioinformatics- and structural homology-based predictions encouraged a closer assessment of whether VNG1056C, VNG1057C, and/or VNG1063H correspond to the sulfotransferase(s) that participate in *Hbt. salinarum* N-glycosylation.

### Deletion of *VNG1056C* or *VNG1057C* affects N-linked tetrasaccharide sulfation

Generating strains deleted of *VNG1056C*, *VNG1057C*, or *VNG1063H* and testing for an effect on N-linked tetrasaccharide sulfation could reveal that one or more of these genes encode the sulfotransferase(s) of the *Hbt. salinarum* N-glycosylation pathway. As a first step in such efforts, reverse transcriptase PCR confirmed that all three genes are transcribed (not shown). Next, strains deleted of *VNG1056C*, *VNG1057C*, or *VNG1063H*, as well as a double-deletion strain lacking both *VNG1056C* and *VNG1057C*, were generated. After confirming the deletion of the gene(s) of interest in each case by PCR and qPCR (Fig. S1), N-linked tetrasaccharide sulfation in each strain was assessed by LC–ESI MS, specifically addressing the N-linked tetrasaccharide decorating Asn-479 in the S-layer glycoprotein-derived ^475^SDAVNSSGGVKDNIDTSDFNQGVSSTSSIR^504^ peptide.

**Fig 1:**
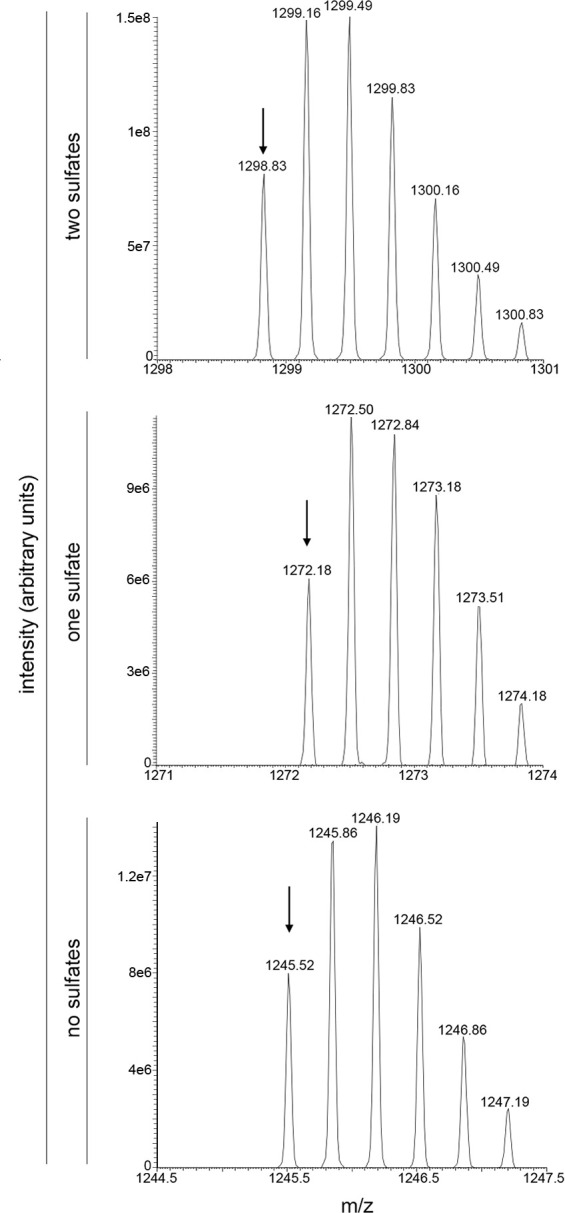
The N-linked tetrasaccharide decorating the *Hbt. salinarum* S-layer glycoprotein is di-sulfated. LC–ESI MS profiles showing peaks at *m/z* 1,298.83 corresponding to the [M + 3H]^3+^ ion of the S-layer glycoprotein-derived Asn-479-containing peptide modified by a tetrasaccharide comprising hexose, hexuronic acid, and two sulfated hexuronic acids (two sulfates; top row), at *m/z* 1,272.18 corresponding to the [M + 3H]^3+^ ion of the same peptide modified by the same tetrasaccharide modified by only one sulfate group (one sulfate; middle row), and at *m/z* 1,245.52 corresponding to the [M + 3H]^3+^ ion of the same peptide modified by the same tetrasaccharide not modified by any sulfate groups (no sulfates; bottom row) from parent strain cells. The arrows depict the peak of interest.

As previously reported (12), the MS profile of the S-layer glycoprotein isolated from the parent strain included a peak at *m/z* 1,298.83 corresponding to the [M + 3H]^3+^ ion of the Asn-479-containing peptide modified by a tetrasaccharide comprising hexose, hexuronic acid, and two sulfated hexuronic acids ([M + 3H]^3+^ ion calculated *m/z* 1,298.82) (Fig. 1, top panel). In addition, the MS profile also included the same peptide carrying the same tetrasaccharide modified by only one sulfate group (peak at *m/z* 1,272.18; [M + 3H]^3+^ ion calculated *m/z* 1,272.15) or by no sulfate groups (peak at *m/z* 1,245.52; [M + 3H]^3+^ ion calculated *m/z* 1245.48) (Fig. 1, middle and bottom panels, respectively).

**Fig 2:**
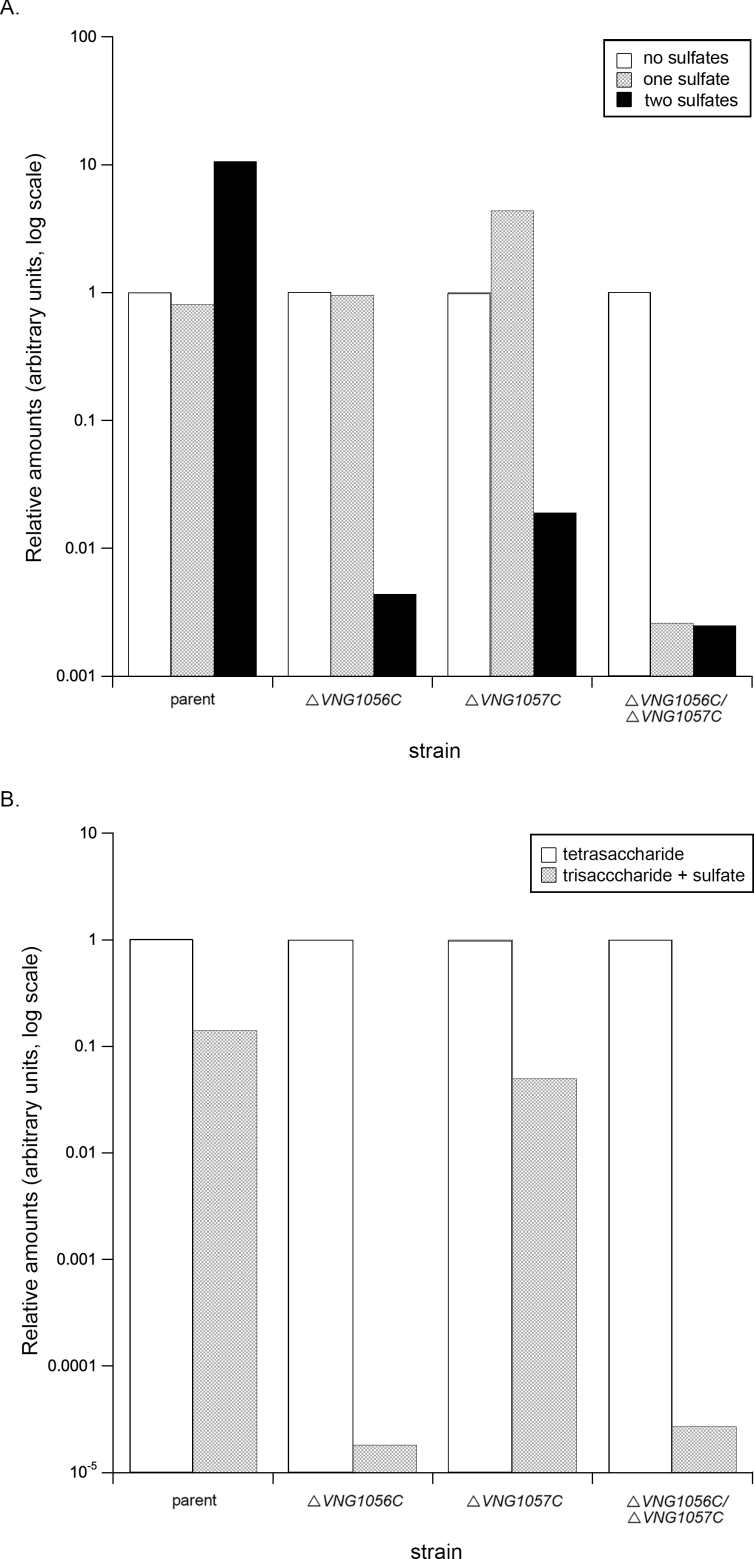
The absence of VNG1056C and/or VNG1057C reduces N-linked glycan sulfation. (**A**) Amounts of non-sulfated (white), mono-sulfated (gray), and di-sulfated (black) N-linked tetrasaccharides in the parent, Δ*VNG1056C*, Δ*VNG1057C*, and Δ*VNG1056C/*Δ*VNG1057C* strains relative to the level of the non-sulfated tetrasaccharide in the same strain normalized to 1.0. The amounts are shown according to log scale. (B) Amounts of the non-sulfated N-linked tetrasaccharide-modified peptide (white) and the mono-sulfated N-linked trisaccharide-modified peptide (gray) in the parent, Δ*VNG1056C*, Δ*VNG1057C*, and Δ*VNG1056C/*Δ*VNG1057C* strains relative to the level of the non-sulfated tetrasaccharide in each normalized to 1.0. The amounts are shown according to log scale. In each panel, a representative of two–five repeats is shown. Average values are presented in Table S1.

To determine the impact of *VNG1056C* or *VNG1057C* deletion on N-linked tetrasaccharide sulfation, as well as the impact of simultaneously deleting both *VNG1056C* and *VNG1057C*, the corresponding MS profiles in each strain were obtained (Fig. S2 to S4). In each case, gene deletion did not fully eliminate tetrasaccharide sulfation but had major effects of the degree of such modification. To compare the relative intensities of peaks representing the S-layer glycoprotein-derived tetrasaccharide-modified peptide bearing one or two sulfates in each strain, the intensities of the relevant peaks were compared to the intensity of the peak corresponding to the non-sulfated tetrasaccharide-modified peak in the same strain normalized to 1.0 (Fig. 2A). In the case of the parent strain, similar amounts of this peptide either bearing no or one sulfate were seen, while some 10-fold more di-sulfate-bearing peptide was noted. In contrast, whereas similar amounts of the peptide either bearing no or one sulfate were seen in the Δ*VNG1056C* strain, 250-fold less of the peptide modified by two sulfates was detected. In cells lacking *VNG1057C*, some four-fold more mono-sulfated peptide than non-sulfated peptide was detected. However, over 50-fold less di-sulfated peptide was seen. When both *VNG1056C* and *VNG1057C* were deleted, over 300-fold less of either sulfated peptide was measured relative to the non-sulfated peptide. Together, the MS results thus argue that both VNG1056C and VNG1057C are sulfotransferases that modify the N-linked tetrasaccharide decorating glycoprotein in *Hbt. salinarum*. Finally, when the relative amounts of the non-, mono- and di-sulfate-modified S-layer glycoprotein Asn-479-containing peptides in the Δ*VNG1063H* strain were similarly assessed, a scenario reminiscent of what was seen with the parent strain was revealed, suggesting that the principal role of VNG1063H is not N-linked tetrasaccharide sugar sulfation (not shown). As such, the primary focus of subsequent studies was on VNG1056C and VNG1057C.

To gain insight into the order in which VNG1056C and VNG1057C act, the MS profiles obtained from the parent and the various deletion strains were examined for the presence of a peak calculated as *m/z* 1,213.49 corresponding to the [M + 3H]^3+^ ion of the Asn-479-containing peptide modified by a trisaccharide comprising hexose, hexuronic acid, and sulfated hexuronic acid. This species corresponds to a truncated version of the di-sulfated tetrasaccharide. Indeed, peaks at *m/z* 1,213.49 or 1,213.50 were detected in the parent strain and in the Δ*VNG1056C*, Δ*VNG1057C*, Δ*VNG1063H* , and Δ*VNG1056C/*Δ*VNG1057C* strains (Fig. S5). However, upon expressing the levels of the peptide modified by this mono-sulfated trisaccharide as a function of the level of the peptide modified by the non-sulfated tetrasaccharide in the same sample normalized to 1.0, it was evident that the relative amounts of peptide bearing the truncated glycan differed considerably in the different mutant strains (Fig. 2B). In the parent strain, there was 1.4 × 10^−1^-fold of the mono-sulfated trisaccharide-bearing peptide than the same peptide presenting the non-sulfated N-linked tetrasaccharide. Similar distributions were seen in the Δ*VNG1057C* and Δ*VNG1063H* strain mass spectrometry profiles (4.95 × 10^−2^- and 5.7 × 10^−2^-fold, respectively). In contrast, far less of the mono-sulfated trisaccharide-bearing peptide was detected in the Δ*VNG1056C* (1.8 × 10^−5^-fold) and the Δ*VNG1056C/*Δ*VNG1057C* (2.7 × 10^−5^-fold) strains relative to the level of the same peptide modified by the non-sulfated tetrasaccharide. As such, it would appear that VNG1056C sulfates the IdoA at position three of the N-linked tetrasaccharide (or possibly its GlcA precursor; see Discussion), while VNG1057C sulfates the terminal GlcA (see Discussion). Moreover, given the mass spectrometry results showing the presence of one sulfate group in the N-linked tetrasaccharide in deletion strains lacking either VNG1056C or VNG1057C (Fig. 2; Fig. S2 to S4), it would appear that each enzyme modifies its target sugar independently of the other.

### Perturbed N-linked tetrasaccharide sulfation has only a small impact on cell motility

**Fig 3:**
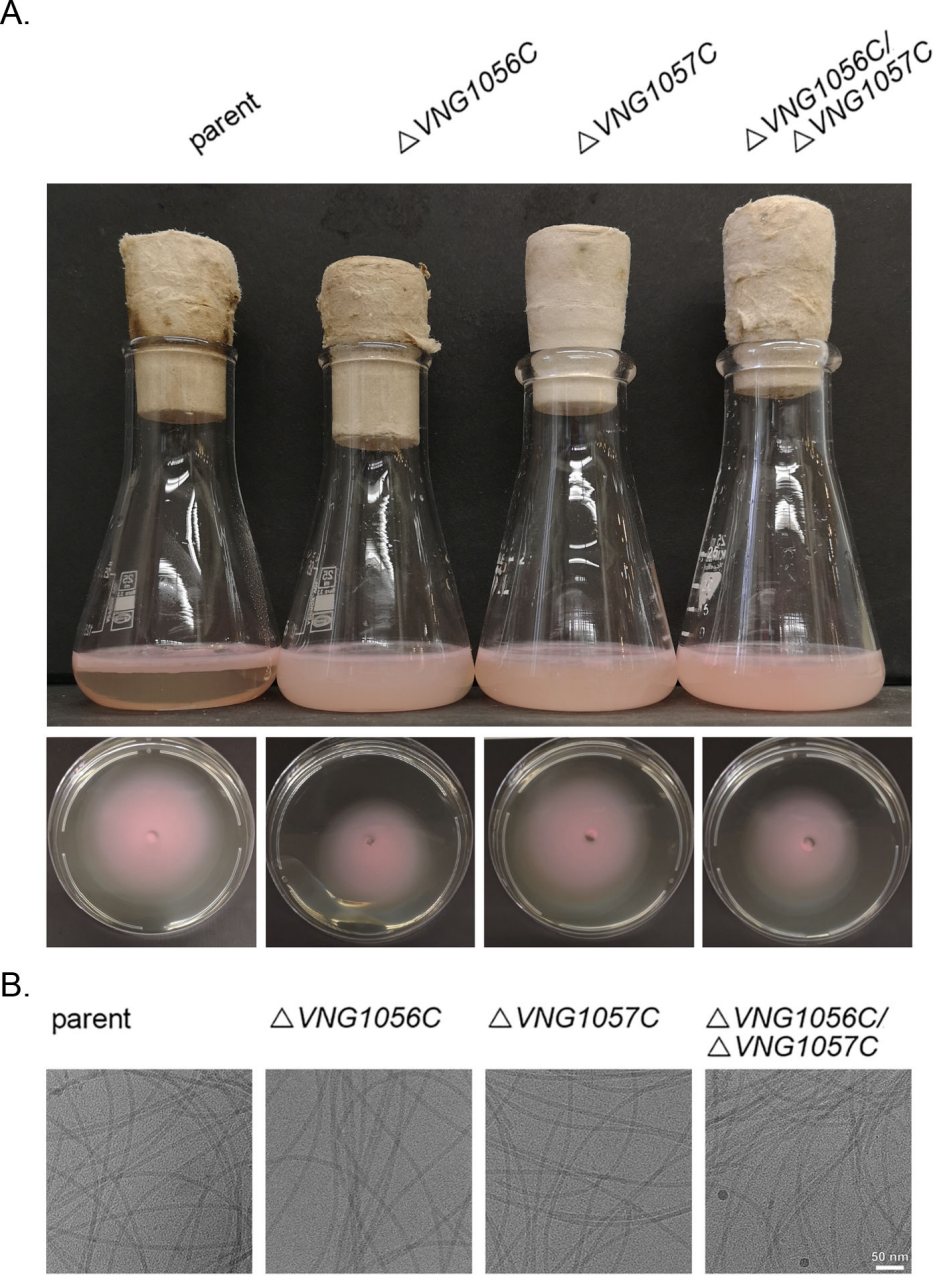
*VNG1056C* or *VNG1057C* deletion has only a minor effect on *Hbt. salinarum* cell motility. (A) Top panels: standing culture of the parent strain shows accumulation of cells at the air/medium interface. In the Δ*VNG1056C*, Δ*VNG1057C*, and Δ*VNG1056C*/Δ*VNG1057C* strains, cells remain more dispersed in the growth medium. Bottom panels: plate motility assays in which 10 µL aliquots of each strain grown to exponential phase were applied to the center of an agar plate, and, 4 days later, the diameter over which the culture had spread was measured. Representative images of four–five repeats per strain are shown. (B) Representative cryo-EM micrographs of archaellum filament samples from the parent, Δ*VNG1056C*, Δ*VNG1057C*, and Δ*VNG1056C*/Δ*VNG1057C* strains are shown.

Cell motility, a process previously shown to be affected by the presence and composition of the N-linked glycan attached to archaellins (7, 8, 10), was next considered in the various deletion strains. As an initial indication of whether deletion of Δ*VNG1056C* and/or Δ*VNG1057C* affected cell motility, stationary phase cultures were left standing on a benchtop overnight. The next day, clear differences were observed in the parent and deletion strain cultures. While cells of the parent strain had migrated toward the surface of the culture medium in the Erlenmeyer flask in which the cells were grown, preferentially near the glass–medium interface, cells of the Δ*VNG1056C*, Δ*VNG1057C*, and Δ*VNG1056C/*Δ*VNG1057C* strains remained much more dispersed throughout the growth medium (Fig. 3A, top panel). In contrast, the standing Δ*VNG1063H* strain culture appeared similar to the parent strain culture (not shown).

To obtain a more quantitative assessment of the effects of the different deletions on cell motility, a plate motility assay was performed in which drops of exponential-phase culture were applied to the center of agar plates, and, 4 days later, the distances over which the different strains had spread were measured (Fig. 3A, lower panels). Whereas the diameter of the area covered by the parent strain culture was 6.59 ± 0.1 cm (average ± standard deviation; *N* = 5 plates), the diameters of the areas covered in the Δ*VNG1056C* and Δ*VNG1056C/*Δ*VNG1057C* strains were 5.33 ± 0.1 cm (*N* = 5) and 5.61 ± 0.09 cm (*N* = 4), respectively. These differences were deemed to be extremely statistically significant according to Student’s *t* test (*P* < 0.0001). Although deletion of *VNG1056C* led to decreased cell motility in this assay, the impact of *VNG1056C* deletion on cell motility was far less than that seen upon deleting genes encoding any of the four glycosyltransferases responsible for the N-linked tetrasaccharide assembly (8, 10). In the case of the Δ*VNG1057C* strain, the diameter of the area covered was 6.52 ± 0.11 cm (*N* = 5), a value not significantly different from what was measured with the parent strain.

Recent findings showed that the absence of the IdoA and/or the GlcA respectively found at the third and terminal positions of the N-linked tetrasaccharide decorated *Hbt. salinarum* archaellins resulted in decreased motility due to archaellum filament bundling (32). To determine whether VNG1056C and/or VNG1057C also contribute to the organization of archaellum filaments, archaella isolated from spent growth media of the corresponding deletion strains were considered using cryo-TEM. The results failed to reveal differences in the extent of archaellum filament bundling in the deletion strains relative to what was seen with parent strain archaellum filaments. While some bundles of twisted archaellum filaments were occasionally seen, most of the archaellum filaments were well separated from each other in all three strains (Fig. 3B). The same result was obtained when isolated archaellum filaments were imaged using negative-stain EM (not shown). At the same time, negative-stain EM imaging of cell-attached archaella did not reveal any differences in archaellum filament bundling among the strains examined. In all strains, cells presented no archaellum filaments (apparently due to filament shearing or detachment), one archaellum filament, or more than two archaellum filaments that were well separated from each other (Table S2; Fig. S6). Only a few cells in some strains, if any, presented bundles of twisted archaellum filaments. Thus, the slight decrease in cell motility observed in the Δ*VNG1056C* and Δ*VNG1056C/*Δ*VNG1057C* strains does not seem to stem from archaellum filament bundling.

The archaellum filament bundling previously observed in the absence of Agl26 or Agl27, the glycosyltransferases respectively responsible for the appearance of IdoA and GlcA at the third and terminal positions of the N-linked tetrasaccharide (8, 32), was proposed as being normally prevented by either steric hindrance or electrostatic repulsion provided by the highly negatively charged tetrasaccharide N-linked to the archaellins that comprise archaellum filaments. Given the limited impact of lost glycan sulfation on cell motility and the lack of archaellum filament bundling, the contributions of IdoA and GlcA sulfation to the overall negative character and size of the N-tetrasaccharide were next considered. Atomic Gasteiger charges were calculated for the di-sulfated tetrasaccharide and mapped onto its van der Waals surface. While some positive charge is found at the reducing end of the tetrasaccharide, where the linking Glc is found, most of the glycan surface is negatively charged, including the surfaces of the IdoA and GlcA respectively found at the third and fourth tetrasaccharide positions. Comparison of the electrostatic potential surfaces of the tetrasaccharide without or with sulfates revealed that, although the sulfate groups contribute to the overall bulkiness and negative charge of the glycan surface, such contributions appear to be minor (Fig. S7).

### The impact of *VNG1056C*, *VNG1057C*, or *VNG1063H* deletion on transcription of the remaining two genes is salt-dependent

**Fig 4:**
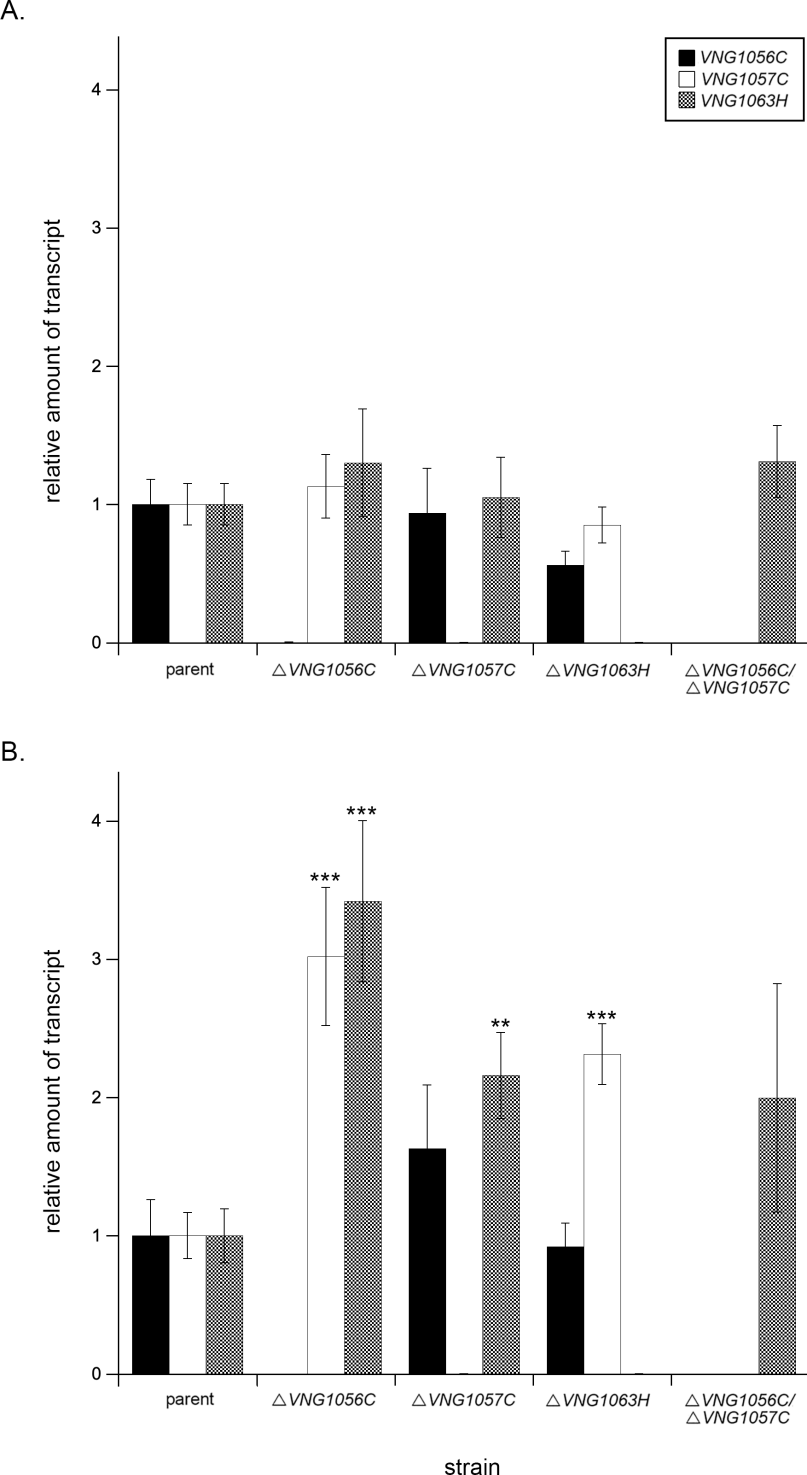
Growth in low salinity leads to up-regulated transcription of *VNG1056C, VNG1057C*, and/or *VNG1063H* in the deletion strains. The levels of *VNG1056C* (black), *VNG1057C* (white), and *VNG1063H* (gray) cDNA reverse-transcribed from mRNA isolated from parent, Δ*VNG1056C*, Δ*VNG1057C*,Δ*VNG1063H*, and Δ*VNG1056C/*Δ*VNG1057C* strains were quantified by qPCR using primers against each gene in cells grown in (**A**) 4.2 M NaCl- or (**B**) 2.9 M NaCl-containing medium. The transcript level of each gene was normalized to that of a housekeeping gene (*VNG0657G*) in the parent strain and corresponding deletion strain. The levels of *VNG1056C*, *VNG1057C*, and *VNG1063H* transcripts were then normalized to the levels measured in the parent strain, taken as 1.0. The values presented represent the average of three biological repeats, each comprising three or four technical repeats, ±standard error of the mean. Where indicated, Student’s *t* test confirmed the significant difference of the level of *VNG1056C*, *VNG1057C*, and *VNG1063H* transcripts relative to the transcript level for each gene in the parent strain in both deletion strains (**P < 0.01; ***P < 0.001). The similar heights of the *Y*-axes in the two panels allow for direct comparison of the results obtained at each salinity.

With bioinformatics- and structural prediction-based support for VNG1056C, VNG1057C, and VNG1063H all being sulfotransferases (see above), in addition to the biochemical support for VNG1056C and VNG1057C serving such roles (Fig. 2), the possibility exists that in the absence of one of the encoding genes, transcription of the others is augmented. Accordingly, transcript levels of two of these genes in the strains lacking the third were measured by qPCR relative to the levels of the housekeeping gene *VNG0657G* (23), and then normalized to the level of transcription of each gene in the parent strain. Such analysis confirmed the absence of transcripts of the deleted gene in each mutant but failed to detect any changes in the transcription of those genes encoding the other assigned or putative sulfotransferases relative to what was seen in the parent strain (Fig. 4A). Similarly, in strains lacking both *VNG1056C* and *VNG1057C*, no increase in the levels of *VNG1063H* transcripts was seen. As such, it seems that *Hbt. salinarum* does not compensate for the absence of VNG1056C, VNG1057C, and/or VNG1063H by augmenting levels of the other apparent sulfotransferases in the different deletion strains.

It is, however, conceivable that *Hbt. salinarum* does modulate the levels of the apparent sulfotransferases remaining in the different deletion strains grown under different growth conditions. To address this possibility, transcript levels of the two remaining genes of interest were considered in cells deleted of the third gene in cells grown in medium containing only 2.9 M NaCl, rather than in 4.2 M NaCl-containing medium in which the cells were normally grown (as portrayed in Fig. 4A). Upon growth at the lower salinity, significantly higher levels of certain transcripts were seen in some of the strains. In the Δ*VNG1056C* strain, transcription of both *VNG1057C* and *VNG1063H* was significantly increased, whereas in the Δ*VNG1057C* strain, enhanced *VNG1063H* transcription was detected. In the Δ*VNG1063H* strain, *VNG1057C* transcript levels were significantly higher than what was seen in the parent strain grown at this lower level of salinity (Fig. 4B).

To assess whether these increases in transcript levels were physiologically significant, the MS analysis described above (Fig. 1) was repeated on the S-layer glycoprotein Asn-479-containing peptide generated from *Hbt. salinarum* cells raised in 2.9 M NaCl-containing growth medium. The relative amounts of mono- and di-sulfated tetrasaccharide-modified peptides, as a function of the amount of the same peptide modified by the tetrasaccharide alone normalized to 1.0, were similar to those obtained with cells raised in a 4.2 M NaCl-containing growth medium (compare Fig. 2A; Fig. S8). As such, it does not seem that the increased transcription of *VNG1057C* and/or *VNG1063H* seen in the different deletion strains grown at the lower salinity was able to compensate for the actions of the missing sulfotransferase.

### VNG1056C is a membrane protein, whereas VNG1057C is a cytosolic protein

Given the demonstrated involvement of both VNG1056C and VNG1057C in *Hbt. salinarum* N-linked tetrasaccharide sulfation, the sub-cellular localization of each protein was next considered. Initially, various predictive algorithms were consulted (https://cctop.ttk.hu/; https://topcons.cbr.su.se/). The consensus of the six algorithms at the former site deemed VNG1056C to be a membrane protein, with residues 21–39 predicted to comprise a transmembrane domain, whereas only one of the six algorithms at the latter site agreed with this prediction. In contrast, VNG1057C was deemed to be a cytoplasmic protein by all of algorithms at both sites, except for one.

**Fig 5:**
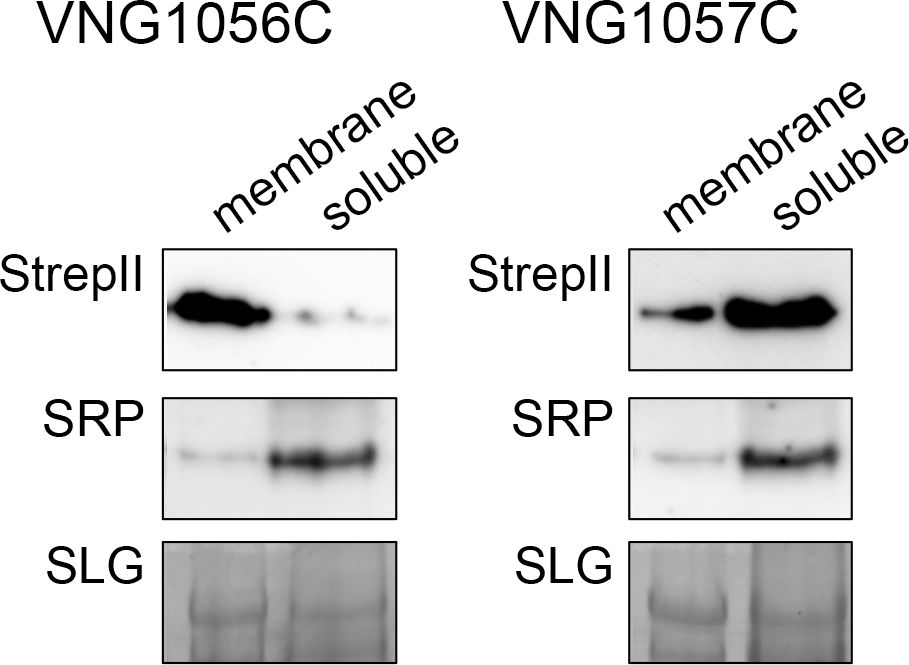
Sub-cellular localization of VNG1056C and VNG1057C. *Hfx. volcanii* cells were transformed to express VNG1056C (left panels) or VNG1057C (right panels) bearing C-terminal StrepII and poly-histidine tags. Following sub-cellular fractionation and SDS-PAGE separation, the membrane (left lanes) and soluble fractions (right lanes) were probed by immunoblot using anti-StrepII antibodies (StrepII; top panels). To control for the efficacy of the fractionation, the same samples were probed with antibodies to *Hfx. volcanii* SRP54 (SRP; middle panels), a known soluble protein (30), or stained with Coomassie blue to detect the S-layer glycoprotein, a known membrane protein (SLG; bottom panels).

To experimentally test these predictions, *Hfx. volcanii* cells were transformed to express versions of either VNG1056C or VNG1057C bearing C-terminal StrepII and poly-histidine tags. Following separation of the soluble and membrane fractions of each transformed strain, immunoblotting was performed using antibodies directed against the StrepII tag. As predicted, VNG1056C was detected in the membrane fractions, while VNG1057C was detected in the soluble fraction (Fig. 5). The efficacy of the sub-cellular fractionation was confirmed when it was shown that SRP54, a known cytosolic protein in *Hfx. volcanii* (30), was restricted to the soluble fraction, whereas the membrane-associated S-layer glycoprotein (36) was mostly found in the membrane fraction.

### Expressing VNG1056C and VNG1057C in *Hfx. volcanii* leads to sulfation of the N-linked pentasaccharide that decorates the S-layer glycoprotein

**Fig 6:**
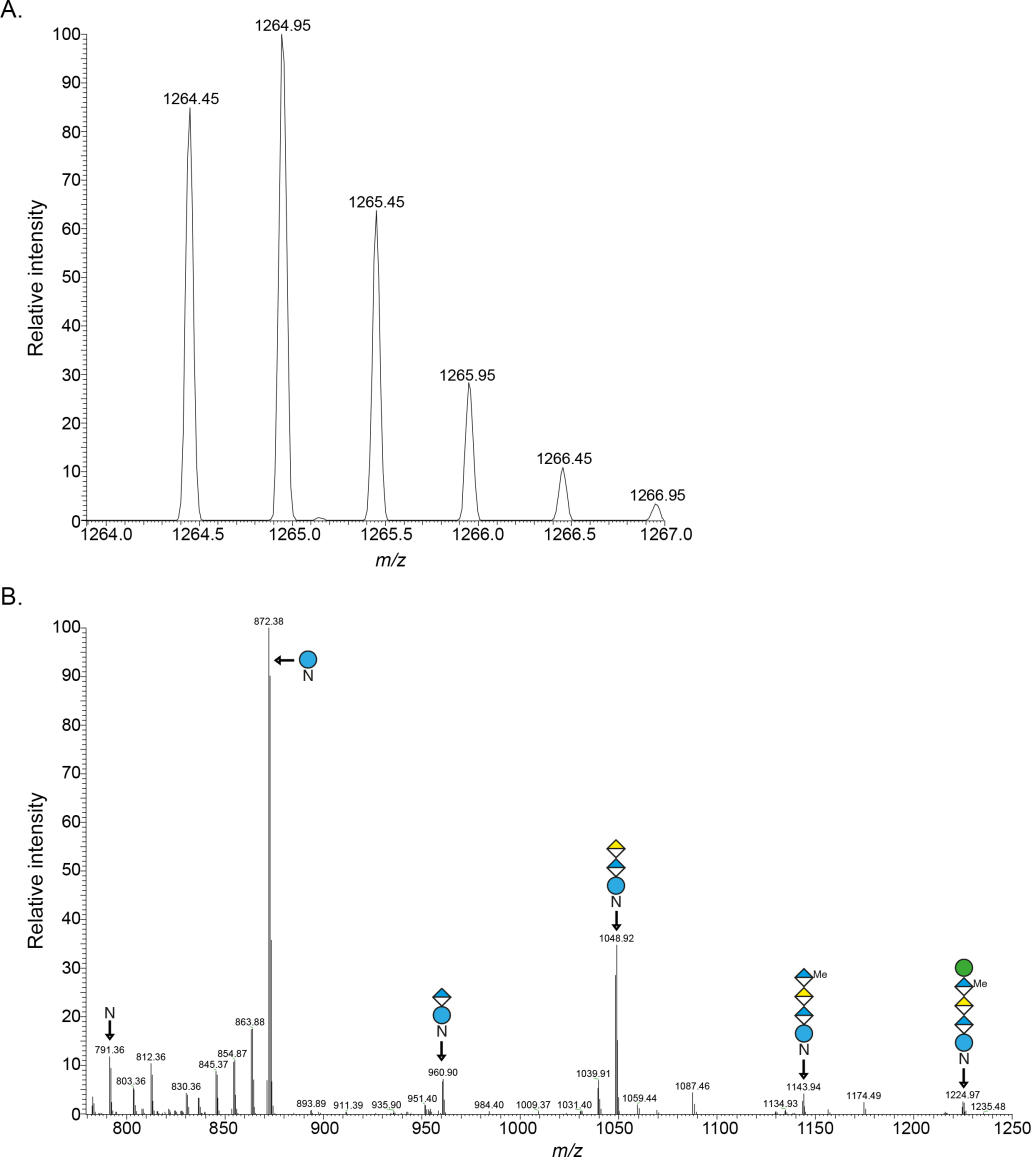
The N-linked pentasaccharide decorating the S-layer glycoprotein of *Hfx. volcanii* cells expressing VNG1056C is sulfated. (A) LC–ESI MS profiles showing a peak at *m/z* 1,264.45 corresponding to the [M + 2H]^2+^ ion of the S-layer glycoprotein-derived Asn-13-containing peptide modified by a sulfated version of the pentasaccharide normally decorating this peptide. (B) MS/MS verification of pentasaccharide sulfation. The sugar contents of the glycans at the relevant peaks are indicated. Glucose is presented as blue circle, glucuronic acid as a blue and white diamond, galacturonic acid as a yellow and white diamond, and mannose as a green circle. Me corresponds to methylation, and N corresponds to the peptide.

In *Hfx. volcanii*, the S-layer glycoprotein is modified by a pentasaccharide comprising Glc, GlcA, galacturonic acid, methylated GlcA, and mannose (37). The availability of *Hfx. volcanii* expressing VNG1056C or VNG1057C bearing a C-terminal StrepII and poly-histidine tag to sulfate sugars comprising this glycan thus offers another route to confirm that these *Hbt. salinarum* proteins are indeed sulfotransferases. Accordingly, LC–ESI MS was performed to address an S-layer glycoprotein-derived peptide containing Asn-13, a residue known to be modified by this pentasaccharide (38, 39). Like that of the parent strain, the MS profiles of cells expressing VNG1056C or VNG1057C both contained a [M + 2H]^2+^ ion peak at *m/z* 1,224.47 corresponding to the pentasaccharide-modified peptide (39) (not shown). However, those profiles from cells expressing VNG1056C or VNG1057C included additional [M + 2H]^2+^ ion peaks not seen in the parent strain profile. *Hfx. volcanii* expressing VNG1056C included [M + 2H]^2+^ ion peaks at *m/z* 1,264.45 (Fig. 6A) and 1,183.45 (not shown) corresponding to the S-layer glycorprotein-derived Asn-13-containing peptide modified by the pentasaccharide and the tetrasaccharide precursor of the pentasaccharide, each modified by a sulfate group. These assignments were supported by MS/MS analysis. The breakdown products obtained from the [M + 2H]^2+^ base peak at *m/z* 1,264.95 included species corresponding to the peptide modified by the complete pentasaccharide (*m/z* 1,224.97) by the tetra-, tri-, di- and monosaccharide pentasaccharide precursors (*m/z* 1,143.94, 1,048.92, 960.90, and 872.38, respectively) and to the non-modified peptide (*m/z* 791.36) (Fig. 6B). The breakdown products obtained from the [M + 2H]^2+^ ion peak at *m/z* 1,183.45 included species corresponding to the peptide modified by the tetra-, tri-, di-, and monosaccharide pentasaccharide precursors and to the non-modified peptide (not shown). When the MS and MS/MS profiles from *Hfx. volcanii* expressing VNG1057C were similarly examined, an [M + 2H]^2+^ ion peak corresponding to the sulfated tetrasaccharide precursor-bearing peptide was identified (Fig. S9). The appearance of MS peaks corresponding to S-layer glycoprotein-derived Asn-13-containing peptides bearing a sulfated glycan in *Hfx. volcanii* cells expressing VNG1056C or VNG1057C lends additional support for these *Hbt. salinarum* proteins being sulfotransferases.

## DISCUSSION

Despite providing the first example of N-glycosylation in Archaea, and, indeed, outside the Eukarya, only recently has progress been made in delineating the pathway used by *Hbt. salinarum* for the assembly of an N-linked tetrasaccharide that decorates glycoproteins in this species. The present report adds further detail to this N-glycosylation pathway with the identification of sulfotransferases responsible for modifying the third and fourth tetrasaccharide sugars. VNG1056C was determined to sulfate the IdoA at tetrasaccharide position three, while VNG1057C was determined to sulfate the tetrasaccharide at the final GlcA.

In addition to identifying sulfotransferases responsible for modifying IdoA and GlcA, this study also provided insight into the order of sugar sulfation. Such insight was gained by considering the levels of the MS peak corresponding to an S-layer glycoprotein-derived peptide modified by only the first three N-linked tetrasaccharide sugars and bearing a single sulfate. This species is likely a breakdown product of the complete tetrasaccharide bearing one or two sulfates since earlier studies addressing cells lacking Agl27, namely, the glycosyltransferase that adds GlcA to the DolP-bound trisaccharide precursor of the complete tetrasaccharide (8), failed to present such a species. This suggests that no sulfation occurs before the complete tetrasaccharide is first assembled. In the present study, three–four orders of magnitude less of the mono-sulfated trisaccharide-modified glycan was bound to the protein in cells lacking VNG1056C or both VNG1056C and VNG1057C relative to the parent, Δ*VNG1057C*, or Δ*VNG1063H* strains. As such, it can be concluded that VNG1056C adds a sulfate to the IdoA at position three of the N-linked tetrasaccharide, while VNG1057C adds a sulfate group to the GlcA at the terminal position of the glycan. This latter assignment is based on the observation that sulfation of one sugar persists in the absence of VNG1056C or VNG1057C, but no such sulfation occurs when both VNG1056C and VNG1057C are lacking and since no decrease in sulfation of the trisaccharide was seen in cells lacking VNG1057C. These findings argue that VNG1056C and VNG1057C sulfate different tetrasaccharide sugars (i.e., the IdoA at tetrasaccharide position three and the terminal tetrasaccharide GlcA, respectively). The distinct tetrasaccharide sugars recognized by each sulfotransferase may explain why VNG1056C is a membrane-linked protein, and VNG1057C is soluble. It is possible to imagine a scenario in which the IdoA sugar of the DolP-linked glycan would be sufficiently close to the membrane for access by VNG1056C. In contrast, the terminal GlcA could be too distant for access by a second membrane-bound sulfotransferase, thus calling for its sulfation by the soluble VNG1057C. In addition to undergoing sulfation, the DolP-bound tetrasaccharide is also methylated on the final GlcA (12). The timing of such methylation relative to sulfation also remains to be defined, although DolP bearing a non-methylated, di-sulfate-modified tetrasaccharide has been described (12), suggesting that methylation is not a prerequisite for sulfation. At the same time, the presently undefined methyltransferase (and the demethyltransferase presumed to exist) would need to be considered in any topological model of tetrasaccharide sugar modification.

In Bacteria and Eukarya, IdoA is generated upon epimerization of GlcA by D-glucuronyl C5-epimerases (40, 41). It is still not known whether the same holds true for Archaea. If GlcA is indeed the precursor of IdoA in *Hbt. salinarum*, then it remains to be determined whether VNG1056C acts on the GlcA precursor or the IdoA product at tetrasaccharide position three. Until the mechanism of IdoA biosynthesis in *Hbt. salinarum* is defined, the possibility of sulfation at the level of the soluble nucleoside-charged putative GlcA precursor of IdoA (or soluble nucleoside-charged IdoA) added to the DolP-bound disaccharide precursor also cannot be discounted. However, the lack of sulfation of the N-linked trisaccharide precursor decorating glycoproteins in the Δ*agl27* mutant, suggesting that tetrasaccharide assembly precedes sulfation (8), argues against VNG1056C sulfating the soluble nucleoside-charged putative GlcA precursor of IdoA (or soluble nucleoside-charged IdoA) added to the DolP-bound disaccharide precursor. As such, it is also reasonable to assume that VNG1057C does not sulfate the soluble nucleoside-charged GlcA added to the DolP-bound trisaccharide precursor of the tetrasaccharide but rather acts on this sugar as part of the tetrasaccharide.

NMR spectroscopy previously revealed IdoA in the N-linked tetrasaccharide to be sulfated at the O-3 position and the terminal GlcA to be sulfated at the O-2 position (9). While GlcA sulfation at the O-2 position has been previously described, such as in eukaryotic chondroitin and heparan sulfate (15), sulfation of IdoA at the O-3 position has yet to be reported other than in the N-linked tetrasaccharide decorating *Hbt. salinarum* glycoproteins. In eukaryotic GAGs, for instance, IdoA is exclusively sulfated at the O-2 position (14, 15). As such, like the incorporation of IdoA into a N-linked glycan, sulfation of IdoA at the O-3 position may also be unique to *Hbt. salinarum* or to Archaea. While the distinct position recognized by each sulfotransferase in the sugar they modify explains why two such enzymes are required, understanding how this is realized will require deciphering the modes of action of VNG1056C and VNG1057C. Still, it should be noted that a basal level of tetrasaccharide sulfation was observed in the absence of both VNG1056C and VNG1057C. At present, neither the agent(s) responsible for such modification nor the positions modified in IdoA or GlcA are known. It should also be noted that the introduction of either VNG1056C or VNG1057C into *Hfx. volcanii*, a haloarchaeal species in which N-glycosylation involves the addition of a non-sulfated pentasaccharide (37), resulted in the sulfation of this glycan, confirming that VNG1056C and VNG1057C are indeed sulfotransferases. Such sulfation occurred at different positions depending on the sulfotransferase expressed. While introduced VNG1056C sulfated the fourth sugar, introduced VNG1057C sulfated the fifth and final sugars. Still, the positions at which each pentasaccharide sugar was sulfated remains to be determined.

Finally, one can ask what purpose N-linked tetrasaccharide sulfation serves. It was previously proposed that *Hbt. salinarum* exploits glycan sulfation as a means to cope with the hyper-saline environment it inhabits, serving to increase the negatively charged character of *Hbt. salinarum* proteins, a property thought to allow the proper folding and function of haloarchaeal proteins (42, 43). Accordingly, sulfation of the N-linked tetrasaccharide decorating glycoproteins in *Hbt. salinarum* but not of the N-linked pentasaccharide attached to glycoproteins in *Hfx. volcanii* was attributed to the former species living in habitats of higher salinity than those inhabited by the latter (44). More recently, it was reported that the absence of the terminal or final two sugars of tetrasaccharide N-linked to *Hbt. salinarum* proteins resulted in a bundling of filaments comprising the archaeal swimming device, the archaellum, with a corresponding loss of motility (32). It was argued that normally, such filament bundling was prevented by either steric hindrance or electrostatic repulsion provided by the highly negatively charged tetrasaccharide N-linked to the archaellins, corresponding to the building blocks of these filaments. The observations that deletion of *VNG1056C* had a relatively limited impact on cell motility, whereas no such impact was noted in the absence of *VNG1057C*, and that deletion of either or both genes did not cause increased archaellum filament bundling, argue that sulfation of tetrasaccharide sugars three and four did not substantially change properties of this N-linked glycan. Indeed, electrostatic potential mapping revealed the sulfate groups added to these sugars made only minor contributions to the overall volume and negatively charged character of the glycan. In the case of VNG1063H, the target it sulfates remains to be identified, assuming VNG1063H to indeed be a sulfotransferase. Given how *VNG1063H* is found embedded within a cluster of genes that encode components of the pathway used to assemble and attach the N-linked tetrasaccharide, the fact that no role for VNG1063H in this pathway was identified is surprising. As *Hbt. salinarum* is known to survive in a variety of growth paradigms (45), the possibility remains that VNG1063H acts in conditions not tested here.

In summary, given the demonstrated roles of VNG1056C and VNG1057C as sulfotransferases in the *Hbt. salinarum* N-glycosylation pathway, they were respectively re-named Agl30 and Agl31 according to the nomenclature used to annotate components of archaeal N-glycosylation pathways (46). The identification of Agl30 and Agl31 not only provides new insight into how this organism performs N-glycosylation but also raises new questions concerning the archaeal version of this universal post-translational modification.

## SUPPLEMENTAL MATERIAL

Supplemental material mbio.03534-24-s0001.docx Supplemental tables and figures.

ASM does not own the copyrights to Supplemental Material that may be linked to, or accessed through, an article. The authors have granted ASM a non-exclusive, world-wide license to publish the Supplemental Material files. Please contact the corresponding author directly for reuse.
