# Supplementary material for: Two different sulfotransferases modify sugars of the N-linked tetrasaccharide decorating Halobacterium salinarum glycoproteins
Source: mBio. 2025 Feb 25;16(4):e03534-24. doi: 10.1128/mbio.03534-24 (PMC11980604; doi:10.1128/mbio.03534-24)
Supplement: Supplemental material — Supplemental tables and figures. [file mbio.03534-24-s0001.docx]

**Supplementary materials**

**Two different sulfotransferases modify sugars of the N-linked tetrasaccharide decorating *Halobacterium salinarum* glycoproteins**

Marianna Zaretsky^1^, Zlata Vershinin^1^, Lihi Erez^1^, Iris Grossman-Haham^1,2^ and Jerry Eichler^1^

^1^Department of Life Sciences, Ben-Gurion University of the Negev, Beersheva, Israel

^2^The Ilse Katz Institute for Nanoscale Science and Technology, Ben-Gurion University of the Negev, Beersheva, Israel

**Corresponding author:** Prof. Jerry Eichler, Dept. of Life Sciences, Ben Gurion University of the Negev, P.O. Box 653, Beersheva 84105, Israel; Tel: +972 8646 1343; Email: jeichler@bgu.ac.il

**Running title:** *Hbt. salinarum* N-linked tetrasaccharide sulfation

**Supplementary Table 1**

|  |  | **Parent** | **Δ*VNG1056C*** | **Δ*VNG1057C*** |
| --- | --- | --- | --- | --- |
| **No sulfates** |  |  |  |  |
|  | average | 1.0 | 1.0 | 1.0 |
|  | std dev | 0 | 0 | 0 |
|  | N | 5 | 4 | 5 |
|  |  |  |  |  |
| **One sulfate** |  |  |  |  |
|  | average | 3.25 | 0.59 | 8.78 |
|  | std dev | 1.79 | 0.55 | 12.89 |
|  | N | 5 | 2 | 5 |
|  | P<0.05 |  | no | no |
|  |  |  |  |  |
| **Two sulfates** |  |  |  |  |
|  | average | 7.54 | 0.017 | 0.025 |
|  | std dev | 3.26 | 0.017 | 0.011 |
|  | N | 4 | 2 | 2 |
|  | P<0.05 |  | yes | yes |

Statistical significance (P<0.5) of the normalized intensity of the peptide modified by a mono-sulfated or di-sulfated glycan in a deletion strain vs. the normalized intensity of the corresponding peptide in the parent strain was determined by t test

**Supplementary Table 2**

| **Strain** | **Parent** | **Δ*VNG1056C*** | **Δ*VNG1057C*** | **Δ*VNG1056C/***  **Δ*VNG1057C*** |
| --- | --- | --- | --- | --- |
| No archaellum filaments | 23 | 16 | 21 | 28 |
| One archaellum filament | 19 | 21 | 13 | 20 |
| >2 separated archaellum filaments | 18 | 19 | 18 | 10 |
| >2 archaellum filaments in a twisted bundle | 4 | 2 | 0 | 0 |
| Number of cells analyzed | 64 | 58 | 52 | 58 |

Examples of cells in each category are provided in Supplementary Fig. 5

**Supplementary figure legends**

**Supplementary Fig. 1 – PCR and qPCR confirmation of gene deletions. A.** To confirm gene deletions and that such deletions did not affect the surrounding genes, PCR amplifications were performed using the indicated primers (see Table 1) and DNA from parent (left in each panel) or *VNG1056C*, *VNG1057C*, *VNG1063H* or *VNG1056C*/*VNG1057C* deletion strains (right in each panel, as indicated) as template. The sizes of the PCR-generated bands are indicated to the left of each panel. **B.** Transcript levels of *VNG1056C* and *VNG1057C* in Δ*VNG1056C*, Δ*VNG1057C* and Δ*VNG1056C*/Δ*VNG1057C* cells, relative to the level of transcripts of the same gene in the parent strain, normalized to 1.0, was determined by qPCR. Each bar represents the average of 9-12 repeats ± standard error of the mean. **C.** Transcript levels of VNG1063H in parent and Δ*VNG1063H* strain cells, normalized to that level measured in the former, taken as 1.0, as determined by qPCR. Each bar represents the average of 10 or 11 repeats ± standard error of the mean.

**Supplementary Fig. 2 – MS profile of an S-layer glycoprotein-derived Asn-479-containing peptide from Δ*VNG1056C* cells.** Absolute amounts of the [M+3H]^3+^ ion of the Asn-479-containing peptide modified by a tetrasaccharide comprising a hexose, a hexuronic acid and two sulfated hexuronic acids (*m/z* 1298.83; top panel), by the same tetrasaccharide modified by only one sulfate group (*m/z* 1272.18; middle panel) or by no sulfate groups (*m/z* 1245.52; bottom panel).

**Supplementary Fig. 3 – MS profile of an S-layer glycoprotein-derived Asn-479-containing peptide from Δ*VNG1057C* cells.** Absolute amounts of the [M+3H]^3+^ ion of the Asn-479-containing peptide modified by a tetrasaccharide comprising a hexose, a hexuronic acid and two sulfated hexuronic acids (*m/z* 1298.83; top panel), by the same tetrasaccharide modified by only one sulfate group (*m/z* 1272.18; middle panel) or by no sulfate groups (*m/z* 1245.52; bottom panel).

**Supplementary Fig. 4 – MS profile of an S-layer glycoprotein-derived Asn-479-containing peptide from Δ*VNG1056C/*Δ*VNG1057C* cells.** Absolute amounts of the [M+3H]^3+^ ion of the Asn-479-containing peptide modified by a tetrasaccharide comprising a hexose, a hexuronic acid and two sulfated hexuronic acids (*m/z* 1298.83; top panel), by the same tetrasaccharide modified by only one sulfate group (*m/z* 1272.18; middle panel) or by no sulfate groups (*m/z* 1245.52; bottom panel).

**Supplementary Fig. 5 - MS profile of an S-layer glycoprotein-derived Asn-479-containing peptide from the various strains addressed in this study.** Absolute amounts of the [M+3H]^3+^ ion of the Asn-479-containing peptide modified by a trisaccharide comprising a hexose, a hexuronic acid and a sulfated hexuronic acid (*m/z* 1213.49) in the (**a**) parent, (**b**) Δ*VNG1056C*, (**c**) Δ*VNG1057C*, (**d**) Δ*VNG1056C*/Δ*VNG1057C* and (**e**) Δ*VNG1063H* strains.

**Supplementary Fig. 6 -** Examples of cells in each category listed in Supplementary Table 1 obtained by negative-stain EM. In that panel showing a cell presenting >2 archaellum filaments in a twisted bundle (lower right panel), the lower left corner shows a zoom-in of the region bounded by a white box.

**Supplementary Fig. 7 - Atomic models of the non-sulfated (left pair) and the di-sulfated (right pair) tetrasaccharide N-linked to *Hbt. salinarum* archaellins.** The stick presentations (left in each pair) show carbon atoms in grey, oxygen atoms in red, and sulfur atoms in yellow. Hydrogen atoms were removed for simplicity. The positions of the sulfate groups in the stick presentation of the di-sulfated tetrasaccharide are indicated with asterisks. In the sphere presentations (right in each pair), spheres, representing the van der Waals radii of each atom, are colored according to atomic Gasteiger charge (red, negative; blue, positive).

**Supplementary Fig. 8 - The relative amounts of mono- and di-sulfated tetrasaccharide-bearing, Asn-479-containing S-layer glycoprotein-derived peptide from strains grown in low-salt conditions.** Amounts of non-sulfated (white), mono-sulfated (grey) and di-sulfated (black) N-linked tetrasaccharides in the parent, Δ*VNG1056C*, Δ*VNG1057C* and Δ*VNG1056C/*Δ*VNG1057C* strains, relative to the level of the non-sulfated tetrasaccharide in the same strain, normalized to 1.0, from cultures grown in 2.9 M NaCl-containing growth medium. The amounts are shown according to log scale.

**Supplementary Fig. 9 – The N-linked tetraasaccharide precursor of the pentasaccharide decorating the S-layer glycoprotein of *Hfx. volcanii* cells expressing VNG1057C is sulfated. A.** LC-ESI MS profiles showing a peak at *m/z* 1183.42.45, corresponding to [M+2H]^2+^ ion of the S-layer glycoprotein-derived Asn-13-containing peptide modified by a sulfated version of the tetrasaccharide precursor of the pentasaccharide normally decorating this peptide. **B.** MS/MS verification of tetrasaccharide sulfation. The sugar contents of the glycans at the relevant peaks are indicated. Glucose is presented as blue circle, glucuronic acid as a blue and white diamond, and galaturonic acid as a yellow and white diamond. Me corresponds to methylation and N corresponds to the peptide.

**Supplementary Fig. 1**

**
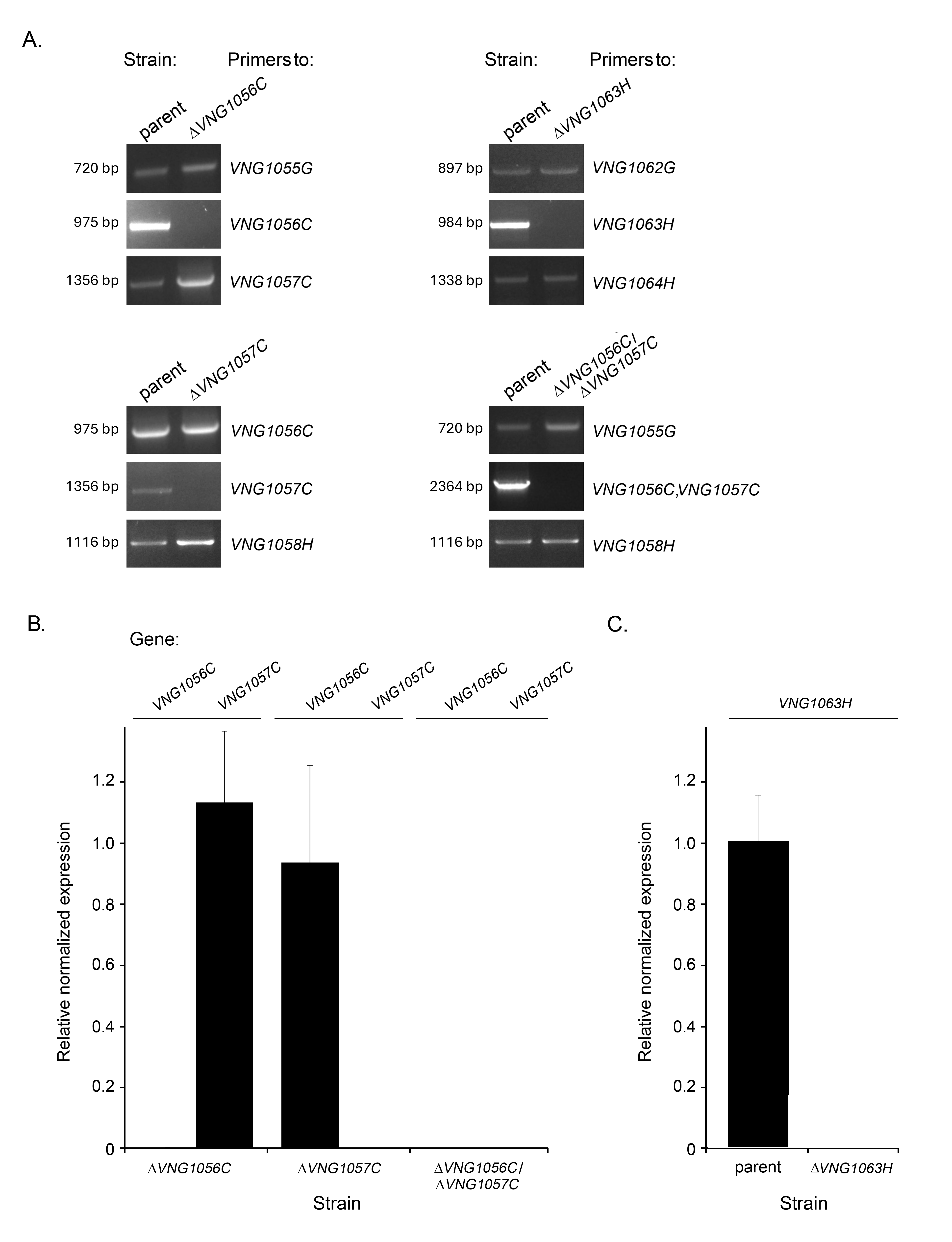
**

**Supplementary Fig. 2**


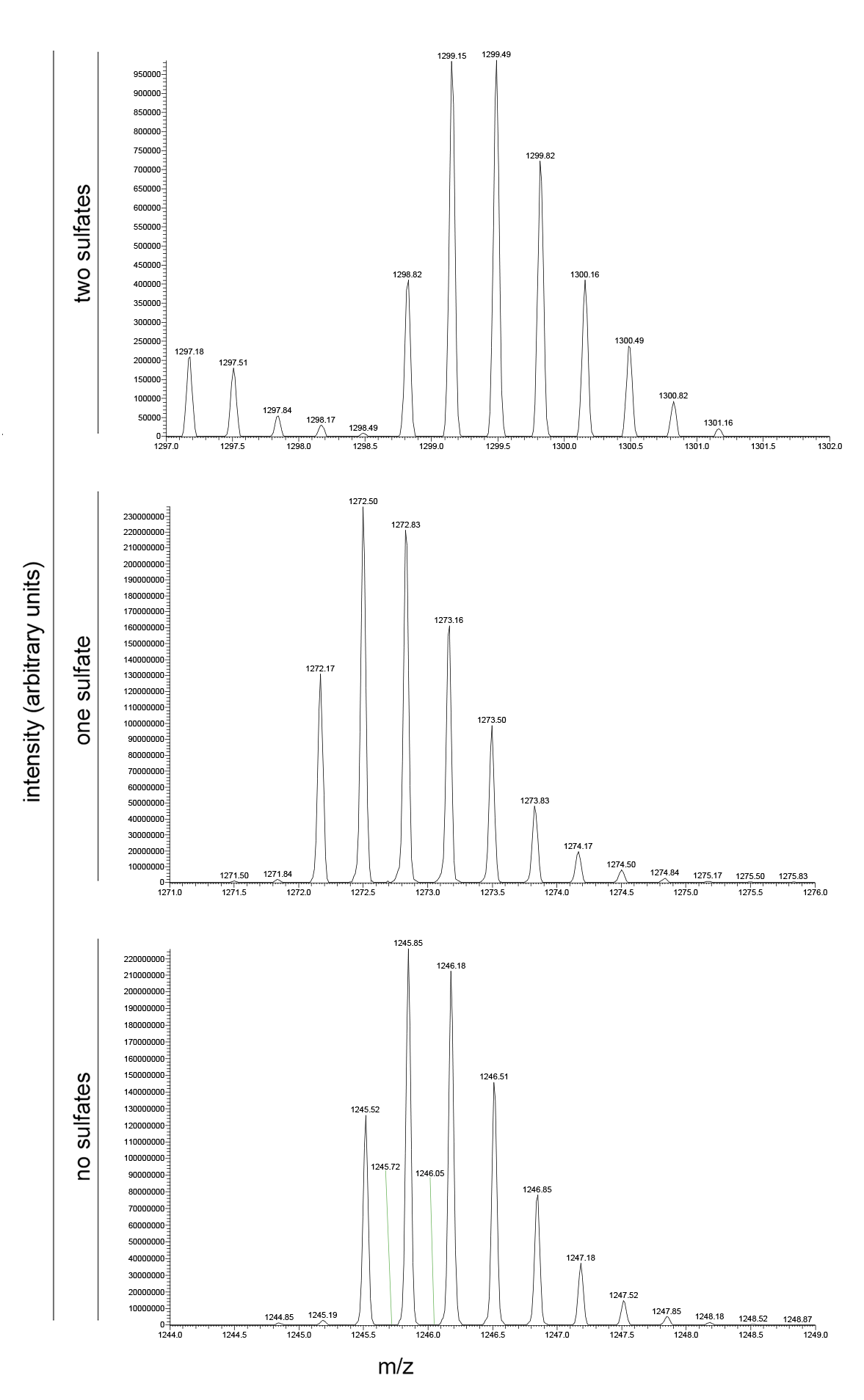


**Supplementary Fig. 3**


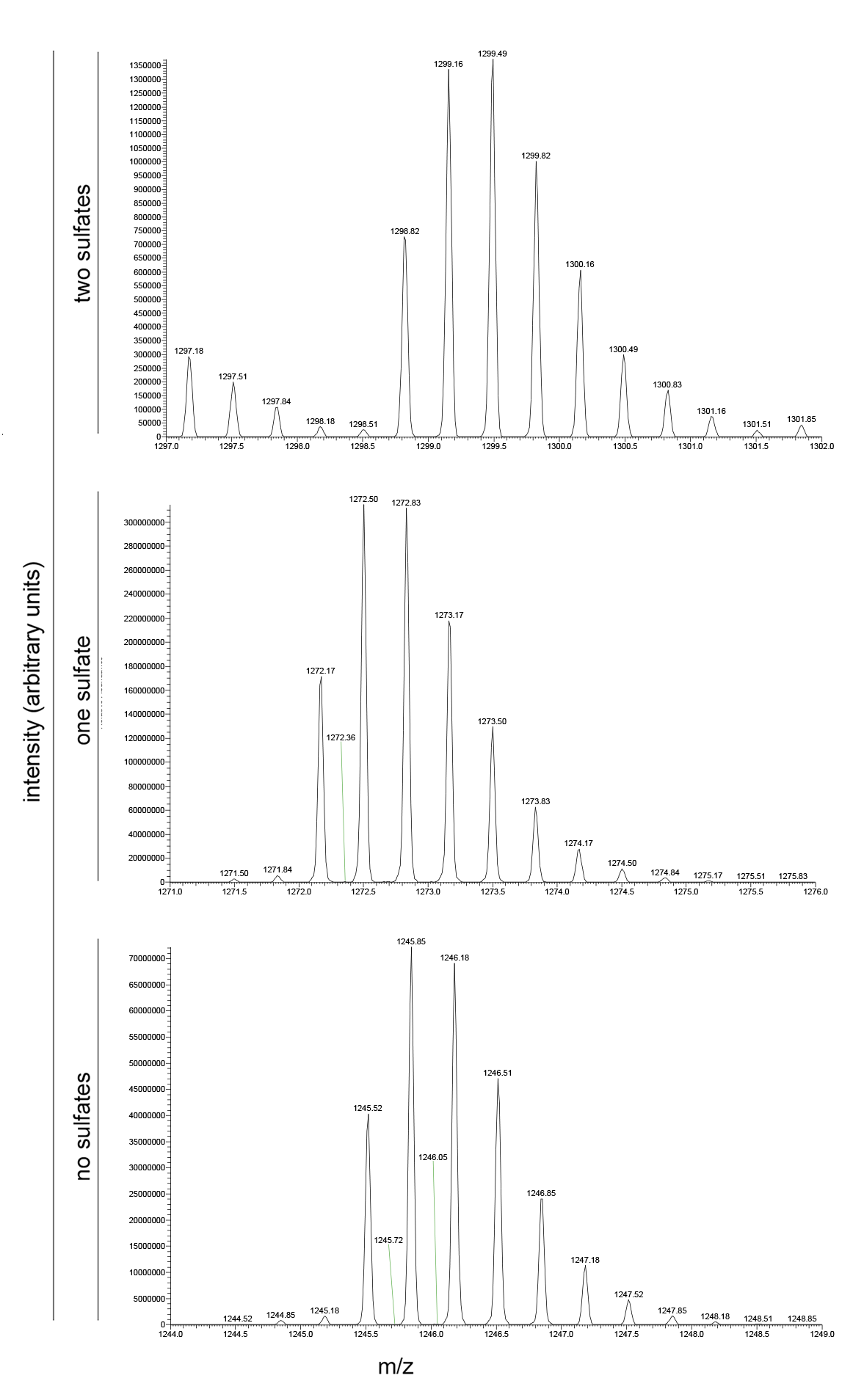


**Supplementary Fig. 4**


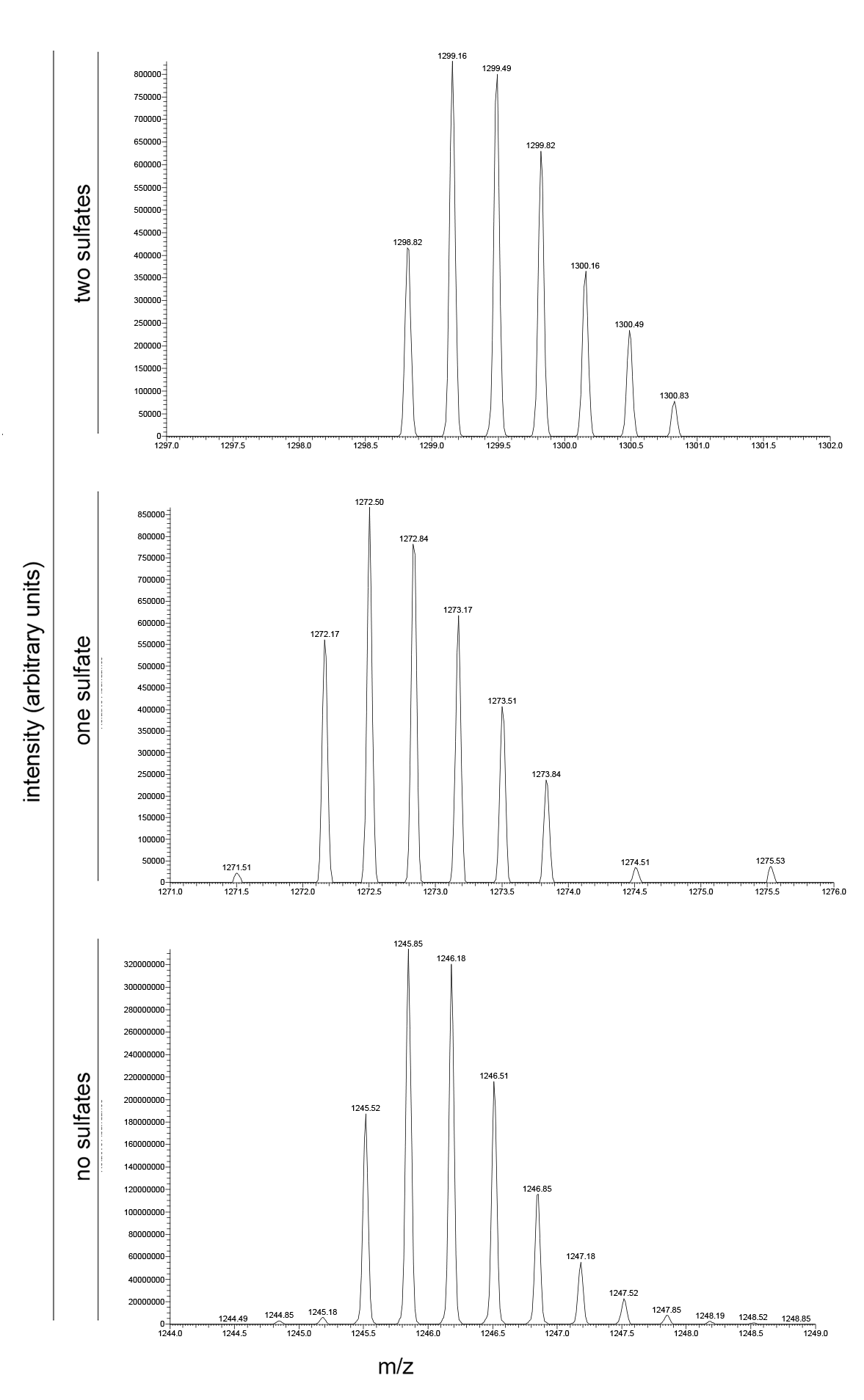


**Supplementary Fig. 5**


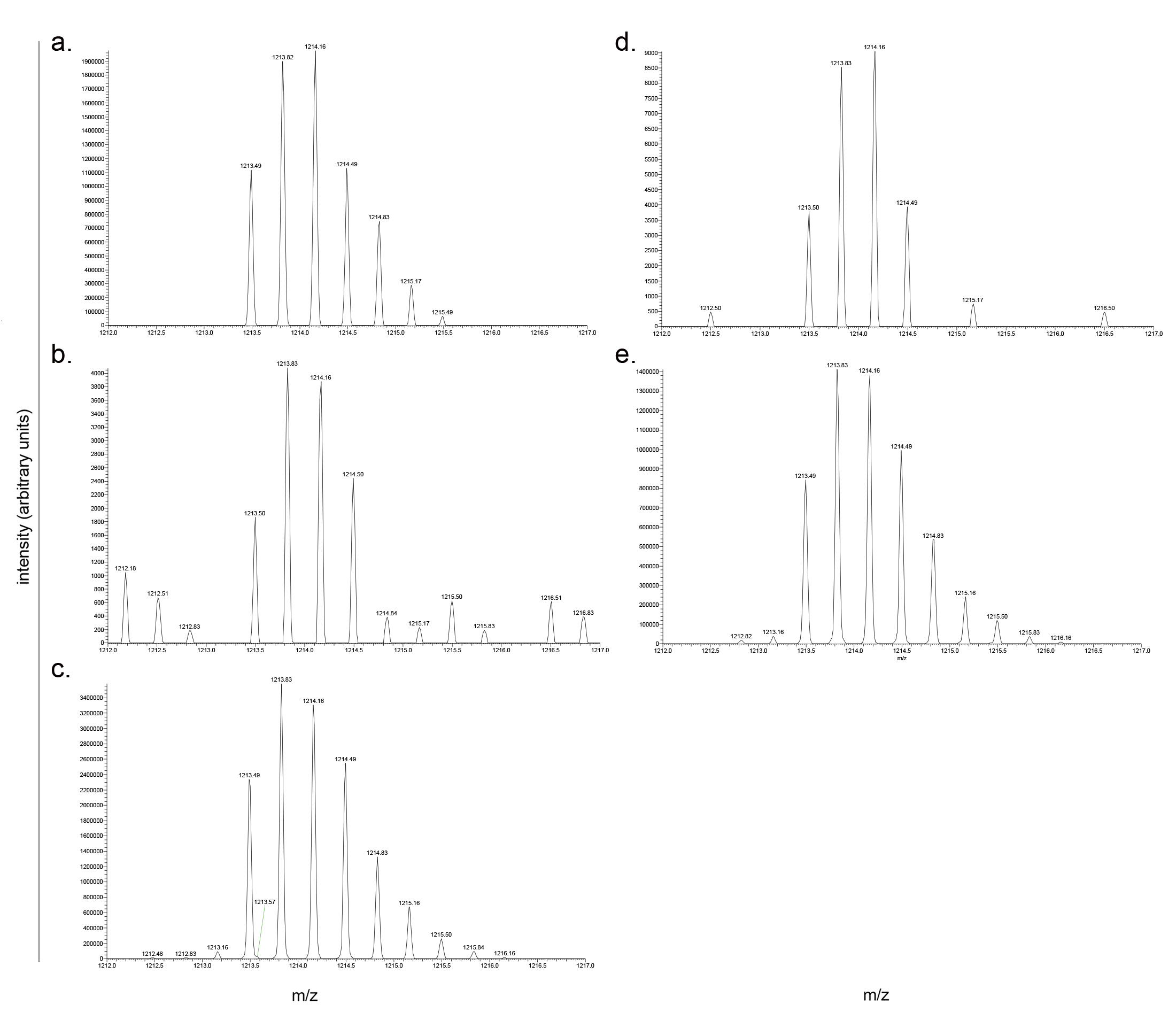


**Supplementary Fig. 6**

**
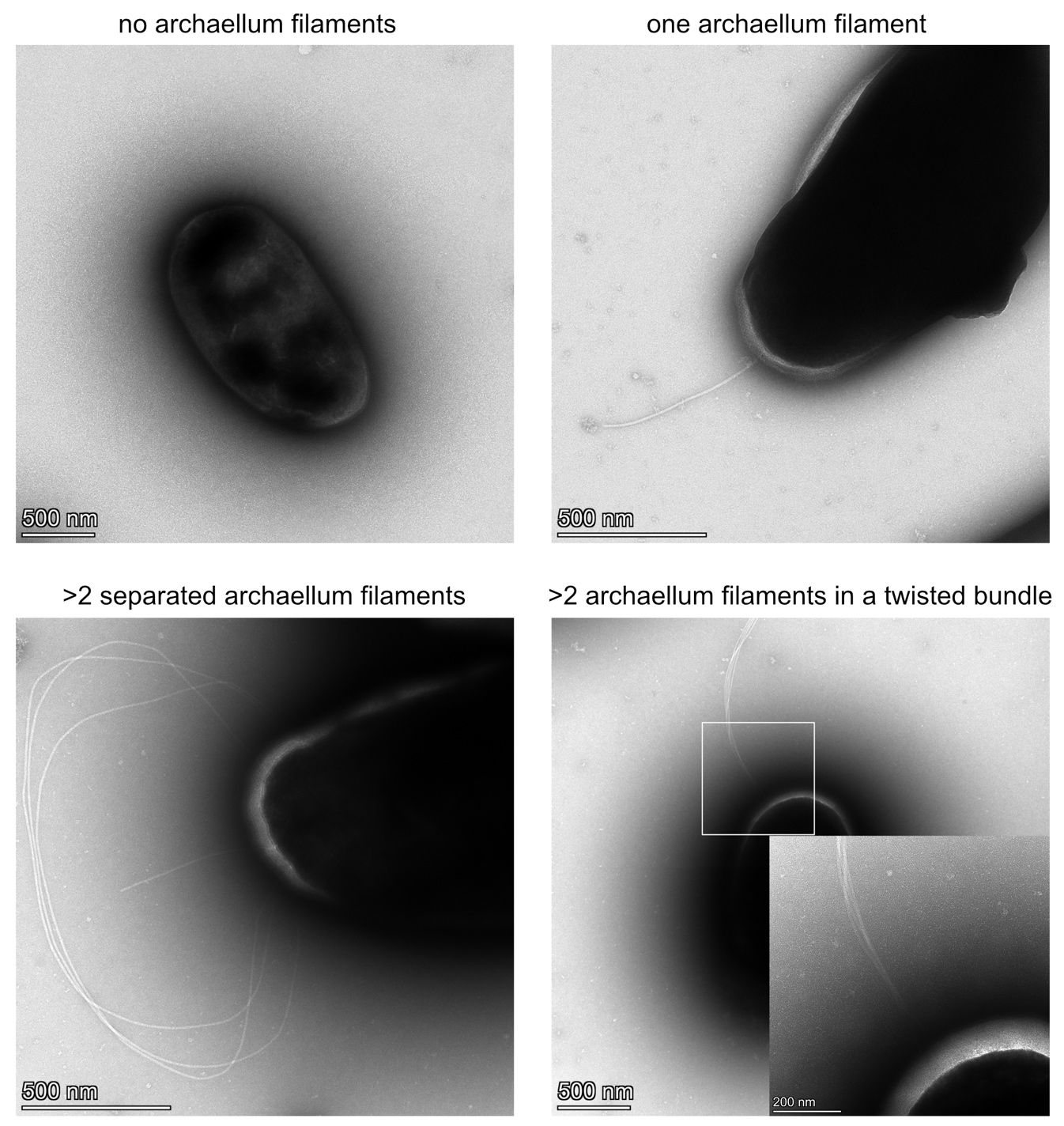
**

**Supplementary Fig. 7**

**
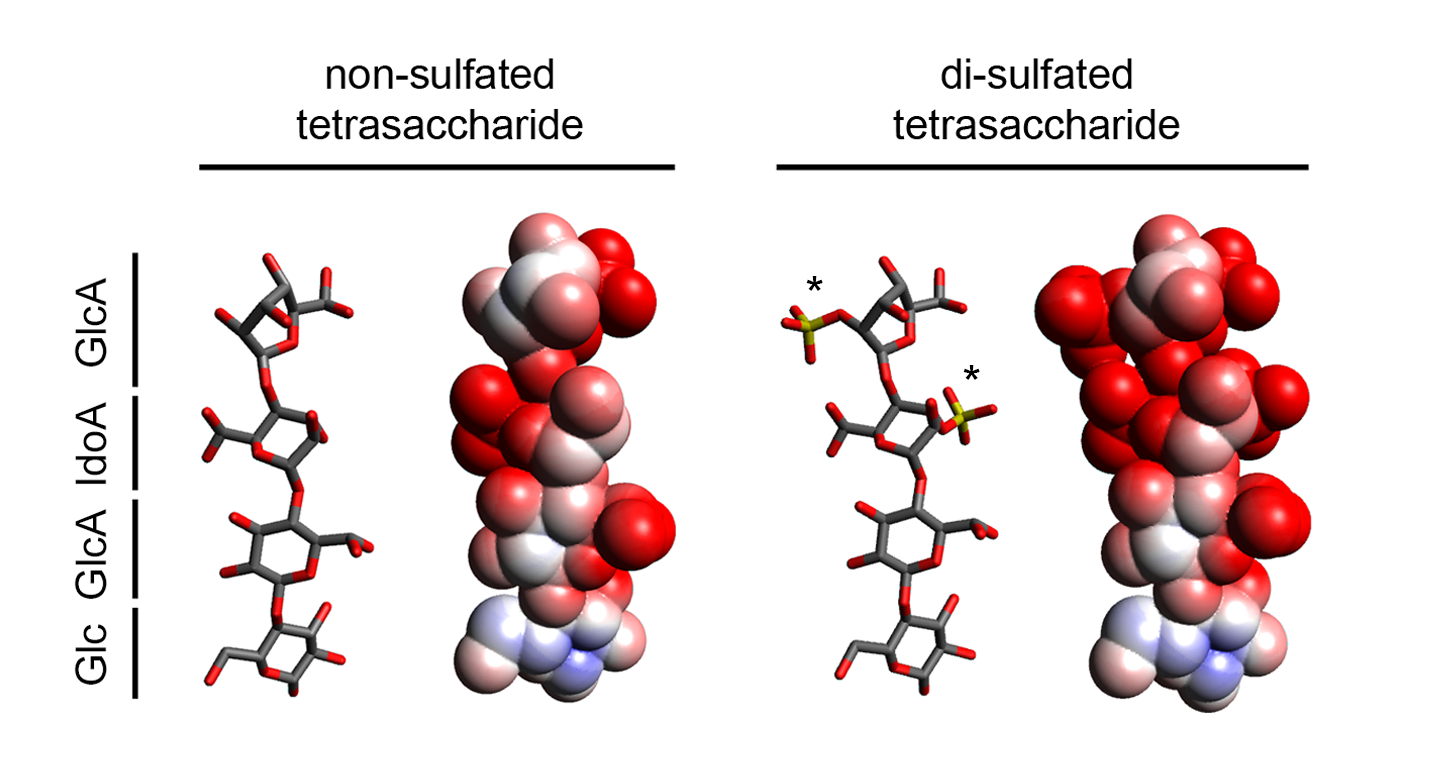
**

**Supplementary Fig. 8**


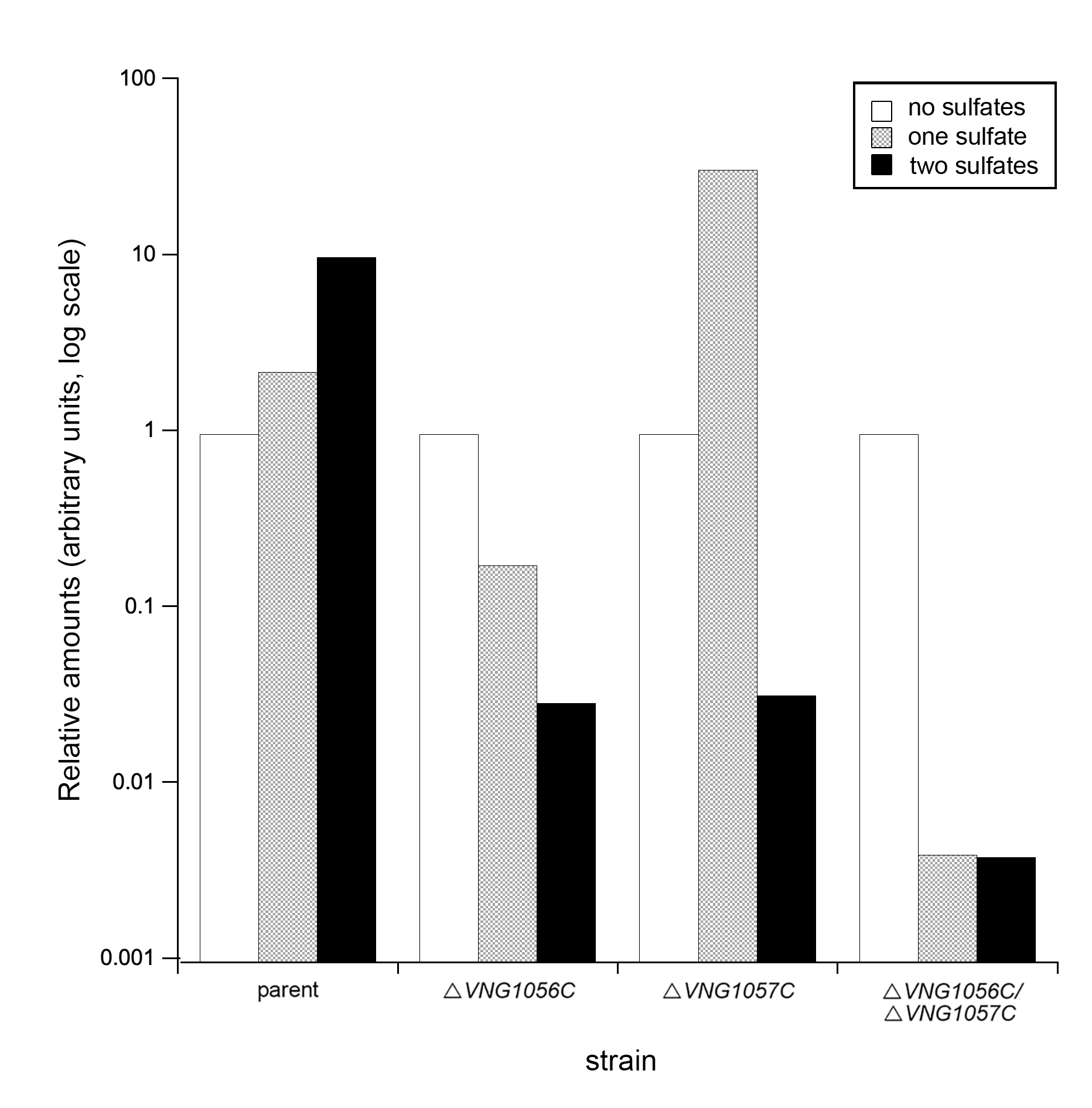


**
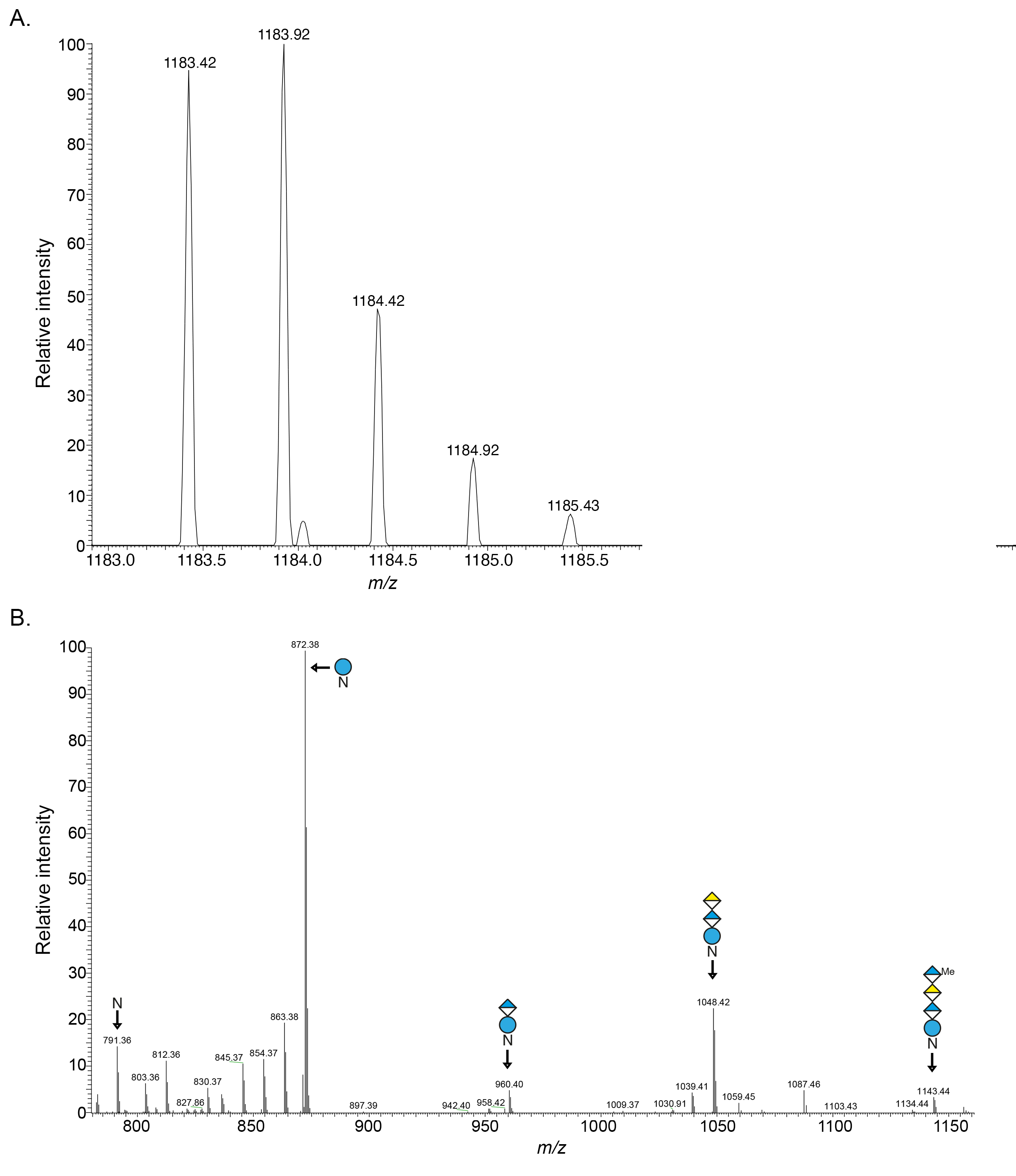
Supplementary Fig. 9**
